# Lower crustal resistivity signature of an orogenic gold system

**DOI:** 10.1038/s41598-021-94531-8

**Published:** 2021-08-04

**Authors:** Graham Heinson, Jingming Duan, Alison Kirkby, Kate Robertson, Stephan Thiel, Sasha Aivazpourporgou, Wolfgang Soyer

**Affiliations:** 1 Department of Earth Sciences, University of Adelaide, Adelaide, SA 5005 Australia; 2 Geoscience Australia, Canberra, ACT 2601 Australia; 3 Geological Survey of South Australia, Adelaide, SA 5001 Australia; 4 WH Bryan Mining and Geology Research Centre, University of Queensland, Brisbane, QLD 4068 Australia; 5 CGG Multi-Physics Imaging, Milan, Italy

**Keywords:** Geology, Geophysics, Tectonics

## Abstract

Orogenic gold deposits provide a significant source of the world’s gold and form along faults over a wide range of crustal depths spanning sub-greenschist to granulite grade faces, but the source depths of the gold remains poorly understood. In this paper we compiled thirty years of long-period magnetotelluric (MT) and geomagnetic depth sounding (GDS) data across western Victoria and south-eastern South Australia that have sensitivity to the electrical resistivity of the crust and mantle, which in turn depend on past thermal and fluid processes. This region contains one of the world’s foremost and largest Phanerozoic (440 Ma) orogenic gold provinces that has produced 2% of historic worldwide gold production. Three-dimensional inversion of the long-period MT and GDS data shows a remarkable correlation between orogenic gold deposits with > 1 t production and a < 20 Ω m low-resistivity region at crustal depths > 20 km. This low-resistivity region is consistent with seismically-imaged tectonically thickened marine sediments in the Lachlan Orogen that contain organic carbon (C), sulphides such as pyrite (FeS_2_) and colloidal gold (Au). Additional heat sources at 440 Ma due to slab break-off after subduction have been suggested to rapidly increase the temperature of the marine sediments at mid to lower crustal depth, releasing HS^−^ ligands for Au, and CO_2_. We argue that the low electrical resistivity signature of the lower crust we see today is from a combination of flake graphite produced in situ from the amphibolite grade metamorphism of organic-carbon in the marine sediments, and precipitated graphite through retrograde hydration reactions of CO_2_ released during the rapid heating of the sediments. Thus, these geophysical data image a fossil source and pathway zone for one of the world’s richest orogenic gold provinces.

## Introduction

**Figure 1:**
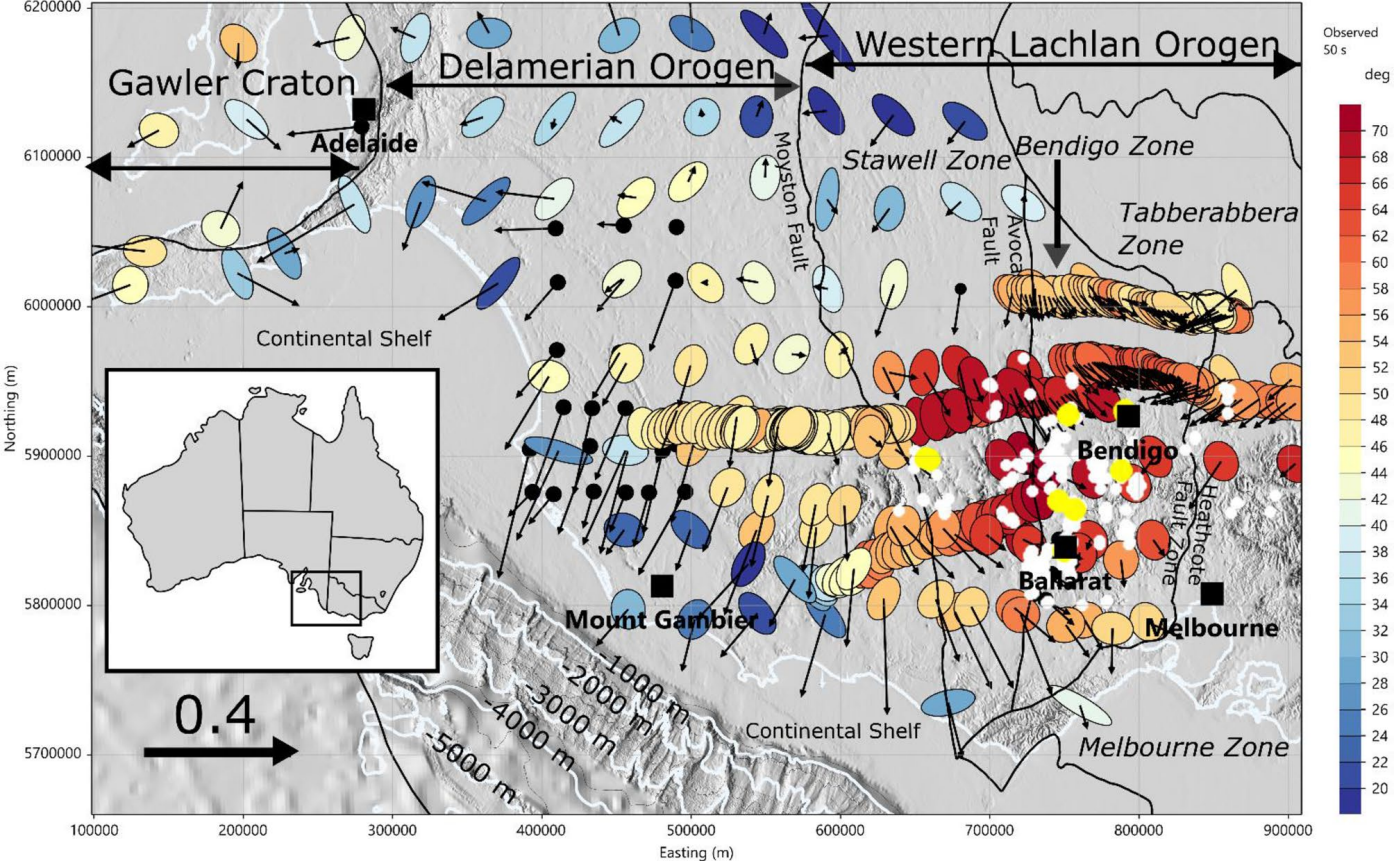
Distribution of MT and GDS sites across south-eastern South Australia and western Victoria, plotted on sun-shaded topography with illumination from the northeast, the coastline and depth contours in 1000 m intervals as solid white lines. Phase tensors at a period of 50 s are colour-filled with the minimum phase: warm-red colours indicate a decrease in resistivity with crustal depth, and cold-blue colours indicate an increase in resistivity. Real (in-phase) induction arrows that in the Parkinson convention point towards conductors are also shown for a period of 300 s. Large yellow circles represent gold mines with production > 1 t; smaller white circles show production < 1 t. Tectonic domains are shown by solid black lines (adapted from^24^). Larger cities are shown (black squares), with Ballarat and Bendigo being centres of historic gold mining. Figure created using CGG Electromagnetics (Italy) Srl Geotools software (version 2.02.12400 www.cgg.com) and Inkscape (version 1.1 inkscape.org). Topography data were obtained from Geoscience Australia Geophysical Archive Data Delivery System under Creative Commons Attribution 4.0 International Licence (portal.ga.gov.au/persona/gadds).

Orogenic gold deposits in upper crustal settings are a significant source of the world’s gold resources, but their origin depth is contentious ^1–5,6,7,8^ The 440 Ma orogenic belt in south eastern Australia is one of the largest global gold provinces and had been widely researched in terms of its structural and geodynamic setting ^9,10–12^ but the 3D crustal architecture is poorly constrained^13^. To provide new insight on the crustal structure beneath this gold province, we have compiled 30 years of broadband and long-period MT and GDS surveys across western Victoria and south-eastern South Australia ^20,21^. Since 2013, high-quality long-period MT data have been collected in the Australian Lithospheric Architecture Magnetotelluric Project (AusLAMP) with site spacing of approximately 55 km^22,23^ that covers all of South Australia and Victoria. After removal of poor-quality sites, 123 long-period MT and GDS sites, 252 broadband MT (mostly with GDS data) and 40 long-period GDS sites were identified as a composite database, covering an area approximately 800 km east–west and 500 km north–south, as shown in Fig. 1.

Three-dimensional inversion of all long-period MT (123 sites) and long-period GDS (40 sites) data, including bathymetry and topography, produced a model that fits the full tensor data to an RMS misfit of 2.1, with error floors of 5% for each impedance element and 0.02 for magnetic transfer functions. A number of different inversions were undertaken, testing: (a) models with and without inversion for distortion; (b) changes in the depth-weighting of smoothness parameters; (c) varying levels of horizontal and vertical regularization; and (d) models with and without near-surface (top 3 km and top 500 m) smoothing. Broadband MT data were not included in the three-dimensional inversion as they have quite a different spatial and frequency sampling^14–17,18,19^. However, we use these data to verify the results from the three-dimensional inversion in the Supplementary Information.

**Figure 2:**
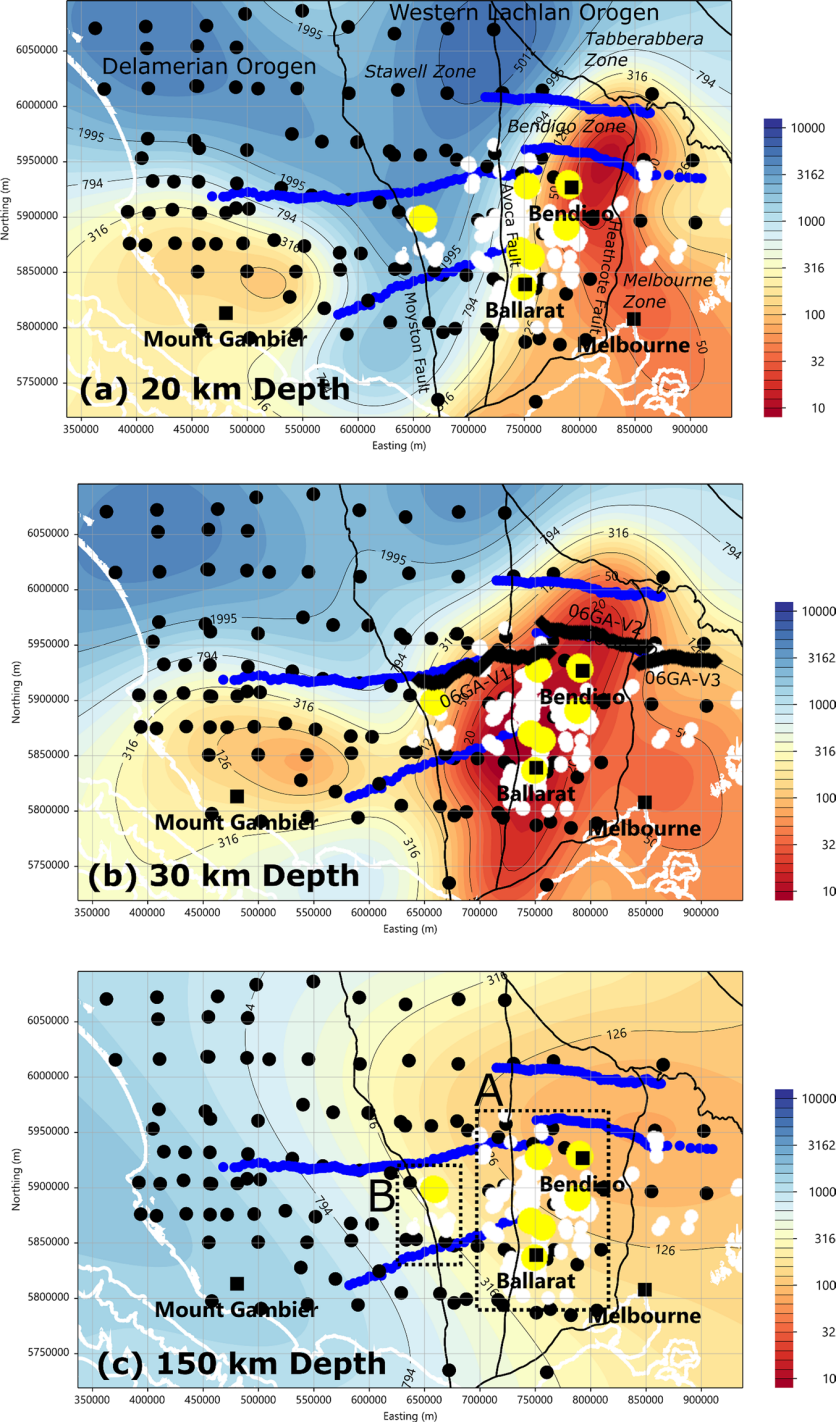
Resistivity depth slices in the lower crust at (**a**) 20 and (**b**) 30 km, and (**c**) near the lithosphere-asthenosphere boundary (~ 150 km). Black circles are long-period MT and GDS observation sites used in the three-dimensional inversion; blue circles are broadband MT transects that were not used in the inversion. Large yellow circles represent gold mines with production > 1 t; smaller white circles show production < 1 t. Solid black lines represent the boundaries of major tectonic elements^24^, and the white lines show coastlines and bathymetry contours at 1000 m depth intervals. The colour scale bar on right side represents resistivity values within the range of 10 to 10,000 Ωm. The image at 30 km shows the location of seismic lines 06GA-V1 to V3. In the 150 km depth slice, box A shows the extent of predominantly orogenic gold deposits, and box B shows predominantly porphyritic and intrusion-related gold deposits, including the Stawell mine. Figure created using CGG Electromagnetics (Italy) Srl Geotools software (version 2.02.12400 www.cgg.com) and Inkscape (version 1.1 inkscape.org). Topography data were obtained from Geoscience Australia Geophysical Archive Data Delivery System under Creative Commons Attribution 4.0 International Licence (portal.ga.gov.au/persona/gadds).

Figure 2 shows three depth slices from the preferred model at lower-crustal depths of (a) 20 and (b) 30 km, and (c) at the lithosphere-asthenosphere boundary depth of ~ 150 km. Details of the 3D inversion parameters are provided in the Supplementary Information and Fig. S1. Additional slices at 5, 10, 40, and 100 km are also shown in Supplementary Information, Fig. S2a–d. At 30 km, there is a strong spatial correlation between major orogenic gold deposits around Ballarat and Bendigo and low-resistivity regions < 20 Ωm.

Such low crustal resistivities in silicate minerals are unlikely to be solely due to temperature, even if minerals are significantly hydrated^25^, so another mechanism to enhance conduction is required^26^. Such additional conduction is generally argued to be graphite^26,27^, magnetite and sulphides^25,28^, or sometimes free fluids^29,30^, which are secondary overprints of the primary crustal materials^31^. Of these competing mechanisms, magnetite and sulphides are generally of negligible volume and are isolated in fresh xenolith granulites^25^, and are unlikely to interconnect over tens of kilometres. Similarly, aqueous fluids are unlikely to be stable and connected over long geological time scales^32^.Thus, the mostly commonly proposed mechanism for widespread electrical conduction at lower crustal depths is graphite^31,27^.

Large et al.^33^ argue that Au deposits in orogenic settings are originally sourced from marine sediments in deep ocean environments that host C, FeS_2_ and potentially colloidal Au. When buried to mid to lower crust depths, and with additional rapid heating from the upper mantle^6,7,9^ such oceanic sediments rich in C and FeS_2_ generate significant amounts of free aqueous sulphur (HS^−^,S^2−^) in a relatively short time frame that acts as the ligand for Au through the following relationship^7^.

$${\text{2FeS}}_{{2}} + {\text{ 2H}}_{{2}} {\text{O }} + {\text{ C }} = {\text{ 2FeS }} + {\text{ 2H}}_{{2}} {\text{S }} + {\text{ CO}}_{{2}}$$

For the case of the Victorian gold province the additional heat source at 440 Ma has been suggested to be due to slab break-off and subsequent mantle upwelling that allowed a rapid introduction of mantle heat into the crust^10^. This mechanism is thought to have occurred along many hundreds of kilometres of a mega-subduction zone off the eastern margin of Gondwana, explaining the widespread occurrences of world-class orogenic and instruction-related gold deposits that were formed simultaneously in the Lachlan Orogen at 440 Ma^10^.

Organic carbon in sediment may be metamorphosed to flake graphite at amphibolite grade conditions at depths of ~ 20–30 km and temperatures ~ 550 °C^34^. In addition, mobilised CO_2_ may be precipitated as graphite^35,36^ either at grain boundaries^27^ or along more permeable zones^37,38^ through retrograde hydration reactions where the host-rock oxygen fugacity (fO_2_^rock^) is below the upper fO_2_ limit of graphite^39^. We argue, therefore, that the low resistivity imaged ~ 30 km depth may be due to graphite. At 20 km depth, the zone of lower resistivity is narrowed and major deposits appear to align along the western margin of the resistivity anomaly, suggesting that the pathway of Au deposits to the surface is controlled by variations in permeability that are expressed as gradients in electrical properties. The resistive western flank may represent a permeability boundary that is structurally aligned with the Heathcote Fault Zone^24,40^. Deposits to the west of the low-resistivity zone at 30 km depth, particularly in the Stawell area, are porphyritic and instruction-related rather than orogenic^11,42,44^.

Figure 2 shows an additional zone of low resistivity of < 300 Ωm centred around the town of Mount Gambier. It is most evident as a separate region in the 20 km depth slice; at 30 km depth the region has slightly lower resistivity (minimum 100 Ωm), but the inherent smoothing of the three-dimensional modelling with increasing depth merges these features. We argue that the cause of this lower resistivity at crustal depths may be due to hotter temperatures associated with the Newer Volcanic Province^43–50^. The most recent volancism (~ 4.5 ka) at Mount Schank, 10 km south of Mount Gambier (volcanism 5 ka), indicates that higher crustal temperatures are still present. For the 20 and 30 km depth slices, an order of magnitude decrease in resistivity (1000 to 100 Ωm) can be explained by thermal anomalies of 100 °C^25^.

At the base of the lithosphere, at a depth of ~ 150 km, there is a resistivity gradient of > 1000 Ωm to 100 Ωm from the southern Delamerian Orogen to the Lachlan Orogen. This trend is also seen in an eastward reduction of P and S-wave velocities^13^ suggesting that there is a step in lithospheric thickness due to higher temperatures beneath the Lachlan orogenic belts.

**Figure 3:**
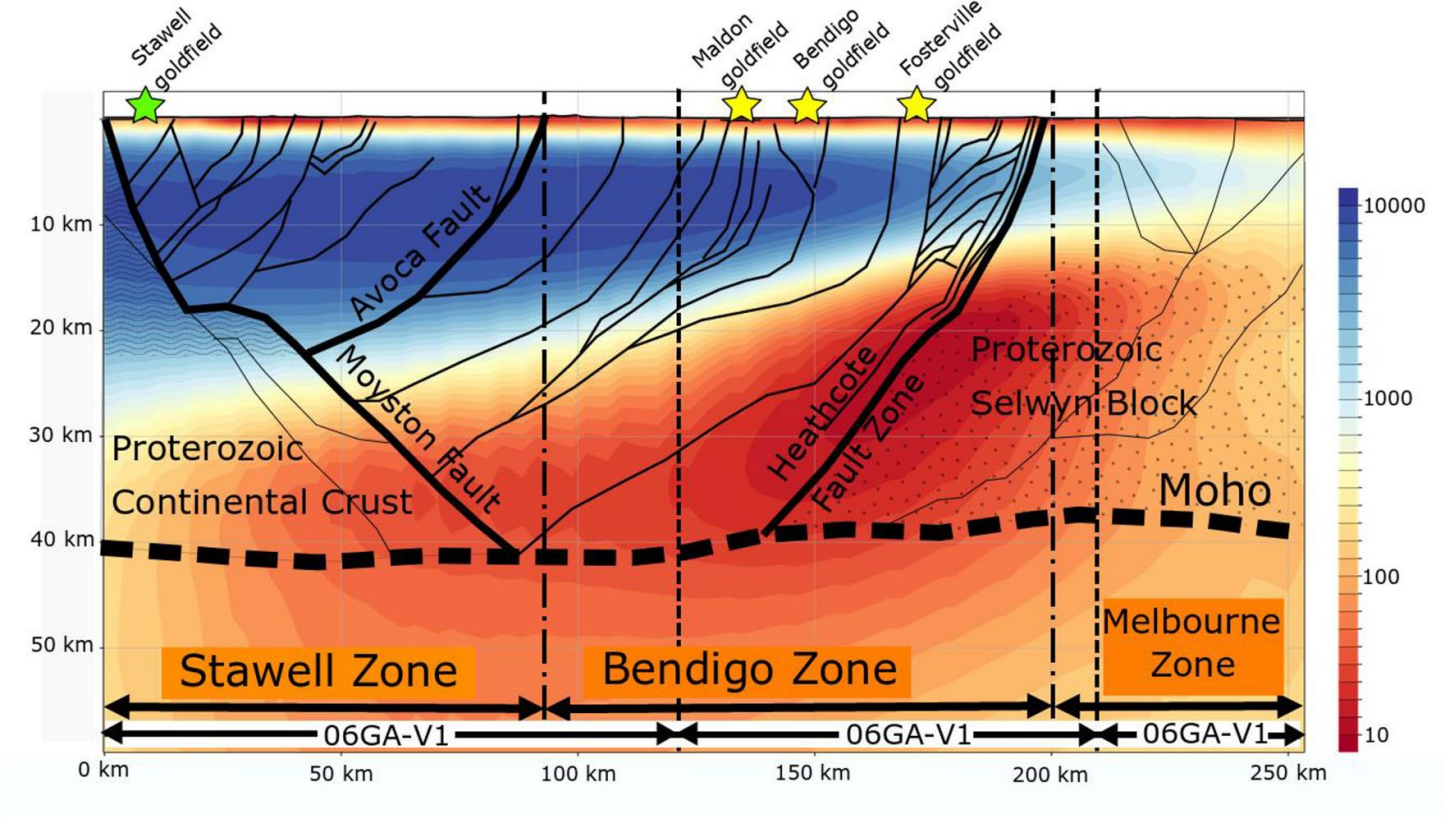
Resistivity section from the three-dimensional model extracted along the three seismic lines 06GA-V1-V3 in Fig. 2b (30-km depth slice), with a simplified structural interpretation^24^. The colour scale bar on right side represents resistivity values within the range of 10 to 10,000 Ωm. Yellow stars represent significant orogenic gold deposits projected on to the seismic lines (Maldon, Bendigo and Fosterville), and the green star represents a porphyritic system (Stawell). Figure created using CGG Electromagnetics (Italy) Srl Geotools software (version 2.02.12400 www.cgg.com) and Inkscape (version 1.1 inkscape.org).

Reflection seismic profiles 06GA-V1 to V3 were collected across the western Victorian goldfields in 2006^12,24^, as shown in Fig. 2b. In Fig. 3 we show a cross-section from the resistivity model along the seismic lines 06GA-V1 to V3 with a structural interpretation derived from the seismic data^24,40^. The resistivity model is a smoothed representation of the geology, but clearly shows that the low-resistivity region (< 20 Ωm) lies near the boundary between the Bendigo Zone and the Selwyn Block in Fig. 3. This low-resistivity region extends from a depth of about 20 km to the seismically defined Moho, and is centred on the west-dipping listric Heathcote Fault Zone, which bounds the Proterozoic Selwyn Block^47^. The east-dipping Moyston Fault that is recognized as the boundary between the Delamerian and Lachlan Orogens^24^ has a less pronounced electrical signature, but delineates the western extent of the low-resistivity region in the lower crust.

The most electrically conductive zone in Fig. 3 is in a broad region of shearing where oceanic mafic crustal elements are highly faulted and stacked above and below the Heathcote Fault Zone^23,24^, representing a zone of enhanced transient permeability during tectonism. The lower resistivity observed along the Heathcote Fault Zone may represent a zone of enhanced graphite deposition from CO_2_-rich fluids^27,37,38^ evolved from tectonically thickened carbon-rich sediments.

## Conclusions

We conclude that the south-eastern Australia orogenic gold deposits have a deep crustal origin. Such gold deposits are spatially correlated with a broad region of lower crust (> 20 km depth) with electrical resistivity of less than 20 Ωm. We argue that this footprint of the source is due to the presence of graphite derived from carbon and pyrite-rich source sediments, from direct metamorphism to flake graphite and precipitated graphite through retrograde hydration reactions of CO_2_ released from the sediments.

## Methods

MT and GDS responses used in the inversion were rotated to 305° (clockwise from geographic N), in line with the 3D mesh orientation. The orientation was primarily chosen to parallel the orientation of the continental shelf and slope to the south. Data were resampled to five per decade over a bandwidth from 10 to 10,000 s, for a total of 16 periods. Error floors of 5% for all tensor impedances and 0.02 for magnetic transfer functions were assigned. Static distortion matrices were also determined from the inversion.

Cell width in the core area is 5 km and the core extended beyond sites by 30 km (6 cells). Lateral padding of 500 km was included, with a growth factor of 1.3. Vertical spacing starts from 100 m at the topographic level, increasing by a factor of 1.06 per cell down to 10 km depth, 1.04 per cell to 100 km, and finally 1.2 until the bottom of the mesh at 800 km. The model includes bathymetry and topography: starting resistivity is a homogenous 200 Ωm, and 0.25 Ωm for sea water. The final model comprised 189 by 123 cells, and 115 layers, to give a total of 2,673,405 free parameters.

## Supplementary Information

Supplementary Information.

## Data Availability

All MT and GDS data are available from the Geoscience Australia (https://doi.org/10.11636/Record.2018.021), the State Government of South Australia (map.sarig.sa.gov.au/) and National Computational Infrastructure (www.nci.org.au/).
