# Supplementary material for: Lower crustal resistivity signature of an orogenic gold system
Source: Sci Rep. 2021 Aug 4;11:15807. doi: 10.1038/s41598-021-94531-8 (PMC8338967; doi:10.1038/s41598-021-94531-8)
Supplement: Supplementary file 1 — Supplementary Information. [file 41598_2021_94531_MOESM1_ESM.pdf]

Lower crustal resistivity signature of an orogenic gold system

Graham Heinson[1*], Jingming Duan[2], Alison Kirkby[2], Kate Robertson[1,3], Stephan Thiel[1,3], Sasha Aivazpourporgou[4], Wolfgang Soyer[5]

[1]Department of Earth Sciences, University of Adelaide, Adelaide, SA 5005, Australia

[2]Geoscience Australia, Canberra, ACT 2601, Australia

[3]Geological Survey of South Australia, Adelaide, SA 5000, Australia

[4]WH Bryan Mining and Geology Research Centre, University of Queensland, QLD 4068, Australia

[5]CGG Multi-Physics Imaging, Milan, Italy

Supplementary Information

Three-dimensional inversion

MT and GDS responses used in the inversion were rotated to 305^0 (clockwise from geographic N), in line with the 3D mesh orientation, as shown in Figure S1. The orientation was primarily chosen to parallel the orientation of the continental shelf and slope to the south. Data were resampled to five per decade over a bandwidth from 10 – 10000 s, for a total of 16 periods. Error floors of 5% for all tensor impedances and 0.02 for magnetic transfer functions were assigned. Static distortion matrices were also determined from the inversion.

Cell width in the core area is 5 km and the core extended beyond sites by 30 km (6 cells). Lateral padding of 500 km was included, with a growth factor of 1.3. Vertical spacing starts from 100 m at the topographic level, increasing by a factor of 1.06 per cell down to 10 km depth, 1.04 per cell to 100 km, and finally 1.2 until the bottom of the mesh at 800 km. The model includes bathymetry and topography: starting resistivity is a homogenous 200 Ωm, and 0.25 Ωm for sea water. The final model comprised 189 by 123 cells, and 115 layers, to give a total of 2673405 free parameters.

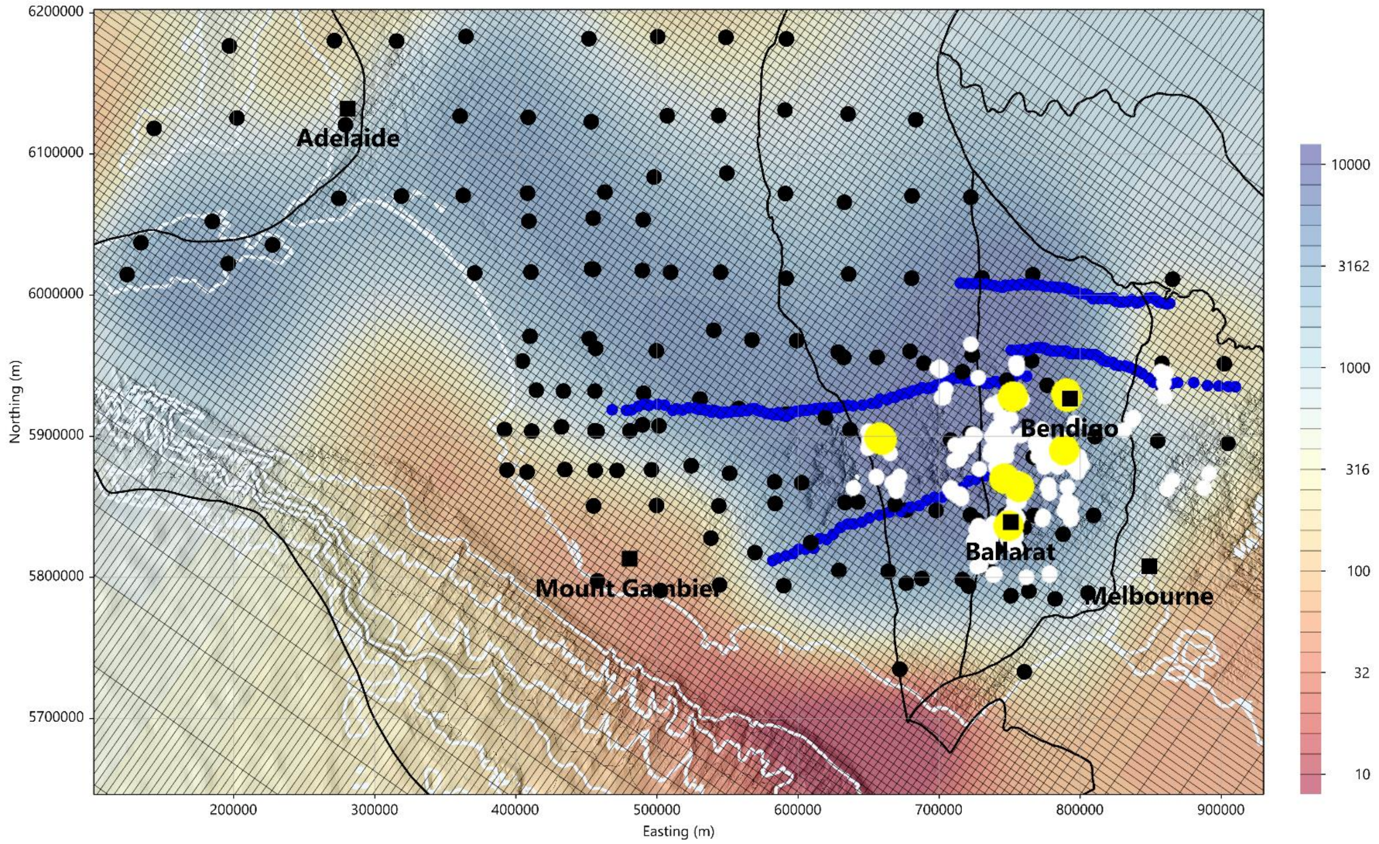

Figure S1: Plan view of the core of the grid, and the 3D inversion at a depth of 10 km. Black dots show long-period MT and GDS sites used in the inversion; blue dots are broadband MT sites that were not included in the inversion due to different spatial-sampling and frequency-bandwidth. White contours are coastline and bathymetry at 1000 m intervals, and black line show major tectonic domains identified in Figures 1 and 2. Black squares and place names show major cities. The colour bar scale ranges from 10 to 10000 Ω.m. Figure created using CGG Electromagnetics (Italy) Srl Geotools software (version 2.02.12400 www.cgg.com) and Inkscape (version 1.1 inkscape.org). Topography data were obtained from Geoscience Australia Geophysical Archive Data Delivery System under Creative Commons Attribution 4.0 International Licence (portal.ga.gov.au/persona/gadds).

Three-Dimensional Model Slices

As for all model studies, a number of different parameterizations were attempted in terms of starting resistivity and smoothing constraints. These include testing: (a) models with and without inversion for distortion; (b) changes in depth-weighting of smoothness parameters; (c) lower horizontal and vertical

regularization to reduce the RM but increase resistivity heterogeneity; and (d) models with and without near-surface (top 3 km and top 500 m) smoothing. The model shown in Figure 2 of the main paper and Figure S2 was chosen as being a balance between data-fit and model smoothness, but the main features identified in the text were present in all inversions.

The following Figure S2 show additional depth slices at (a) 5 km, (b) 10 km, (c) 40 km, (d) 60 km and (e) 100 km. In the 5 km and 10 km slices, we also show sediment thickness in metres of the marginal Otway Basin. There is a clear correlation between the region of low-resistivity in the top 10 km (<10 Ω.m) and the porous sediments, particularly in the deepest parts of the basin.

The lower crustal resistivity anomaly coincident with the orogenic gold deposits is clearly evident at 40 km, but sub-Moho at 50 km the anomaly diminishes in significance with a minimum resistivity of about 50 Ω.m compared with 20 Ω.m at 40 km. It is probable that the uppermost mantle is more resistive but there is low resolution beneath the lower-crustal conductor.

As most of the AusLAMP data have quality estimates to periods of 10000 s, the model has resolution over lithospheric scale-lengths. At a depth of 100 km, the models show a gradient in resistivity from the south west (~1000 Ω.m) to the north east (~100 Ω.m), which may be accountable by changes in temperature of a hundred degrees. The trend reflects the changes from Delamerian to Lachlan aged lithosphere, with younger and hotter lithosphere to the north east. The mechanism to produce intra-plate volcanism of the Newer Volcanic Province has been potentially linked to edge-driven convection processes[1] at a step in the lithosphere rheological properties, and we speculate that changes in resistivity could be a proxy for such a step.

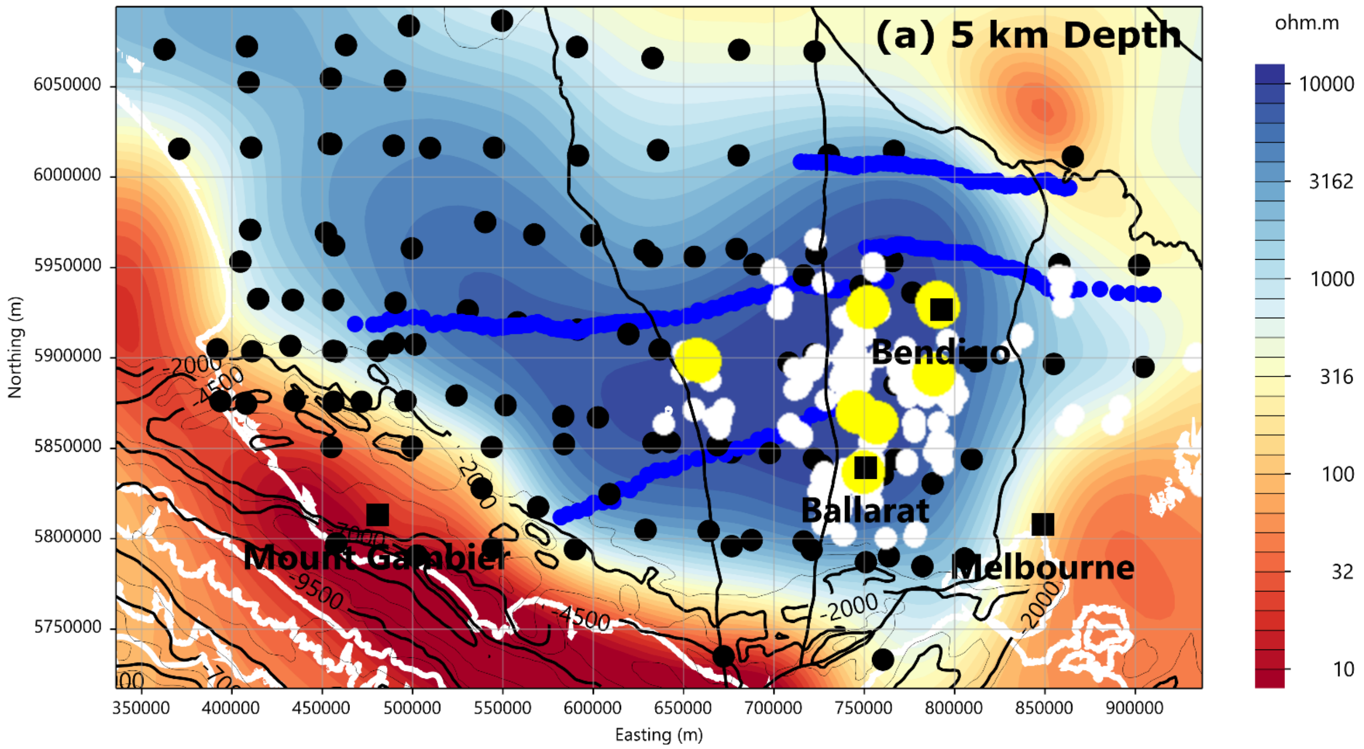
(a) 5 km Depth
ohm.m
10000
3162
1000
316
100
32
10
Northing (m)
6050000
6000000
5950000
5900000
5850000
5800000
5750000
Easting (m)
350000
400000
450000
500000
550000
600000
650000
700000
750000
800000
850000
900000
Bendigo
Ballarat
Mount Gambier
Melbourne
-2000
-4500
-9500
-7000

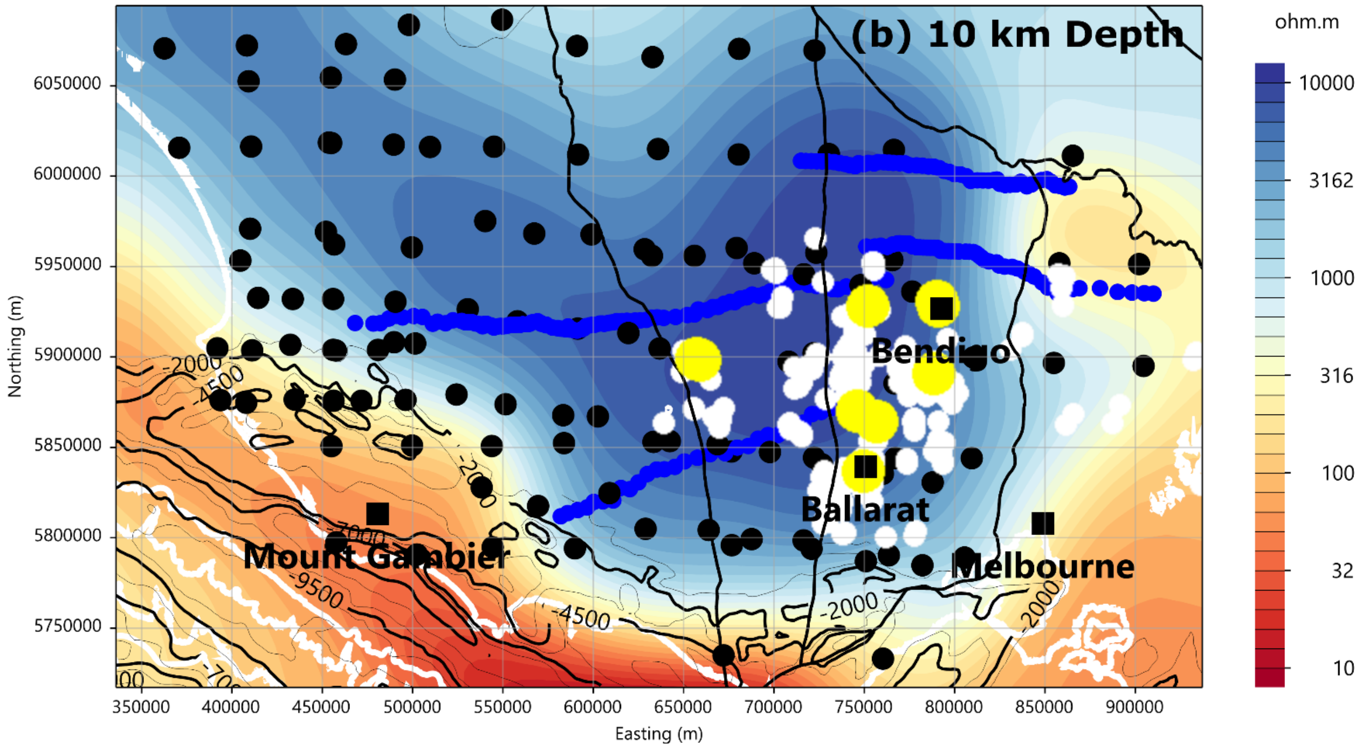
(b) 10 km Depth
ohm.m
10000
3162
1000
316
100
32
10
Northing (m)
6050000
6000000
5950000
5900000
5850000
5800000
5750000
Easting (m)
350000
400000
450000
500000
550000
600000
650000
700000
750000
800000
850000
900000
Bendigo
Ballarat
Mount Gambier
Melbourne
-2000
-4500
-9500
-7000

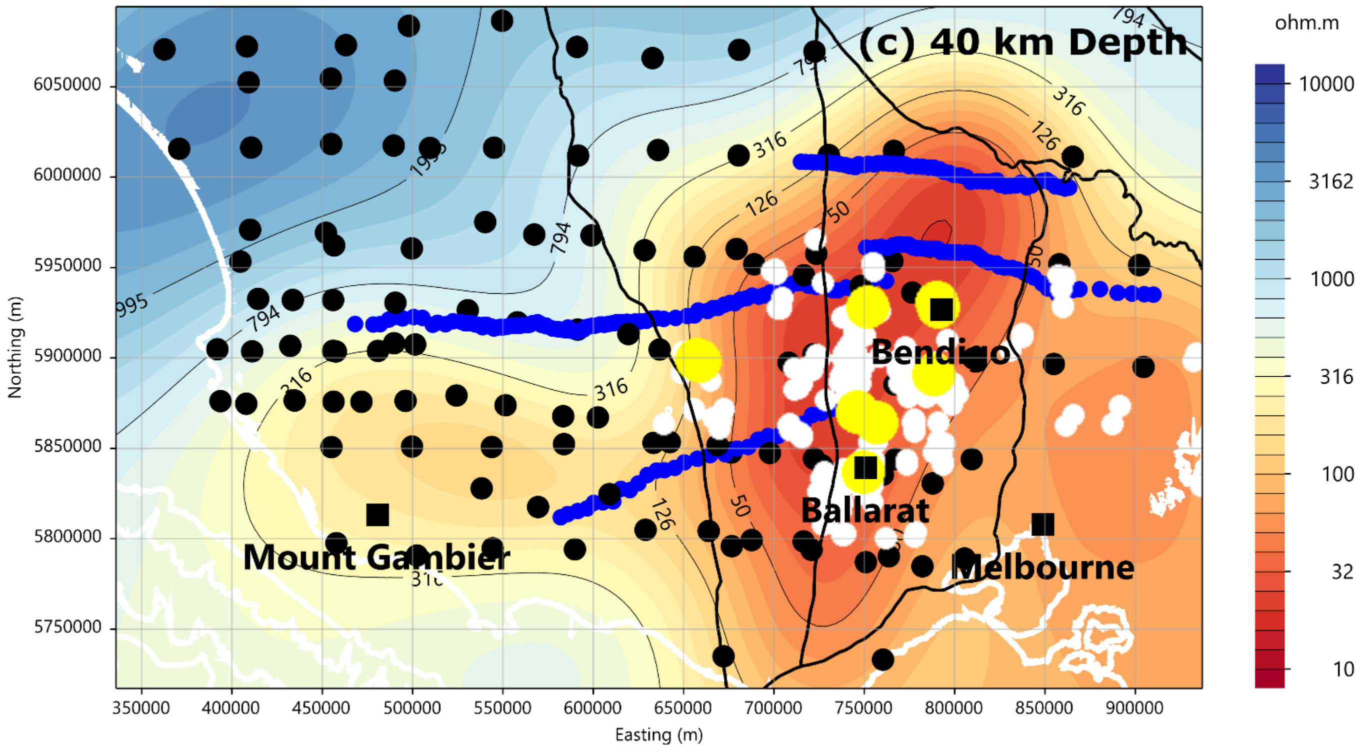
(c) 40 km Depth
ohm.m
10000
3162
1000
316
100
32
10
Bendigo
Ballarat
Mount Gambier
Melbourne
Northing (m)
Easting (m)
6050000
6000000
5950000
5900000
5850000
5800000
5750000
350000
400000
450000
500000
550000
600000
650000
700000
750000
800000
850000
900000

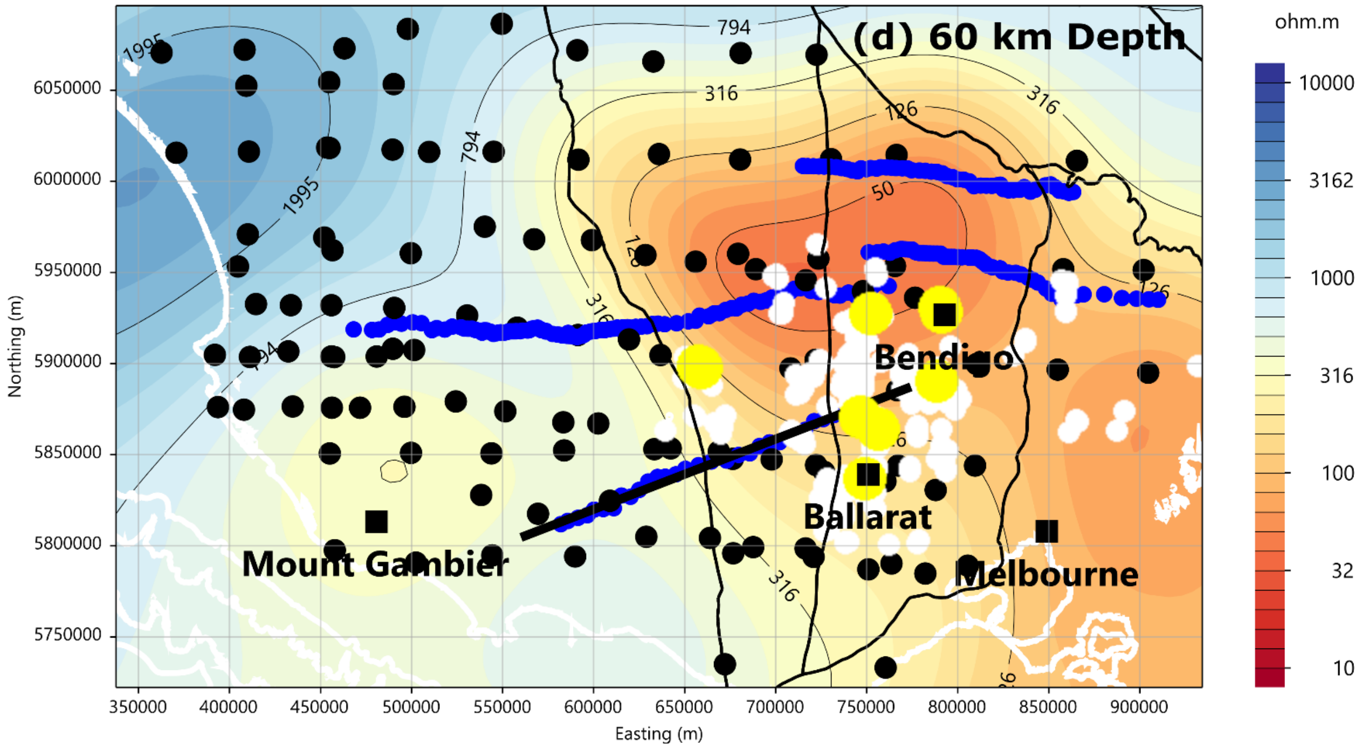
(d) 60 km Depth
ohm.m
10000
3162
1000
316
100
32
10
Bendigo
Ballarat
Mount Gambier
Melbourne
Northing (m)
Easting (m)
6050000
6000000
5950000
5900000
5850000
5800000
5750000
350000
400000
450000
500000
550000
600000
650000
700000
750000
800000
850000
900000

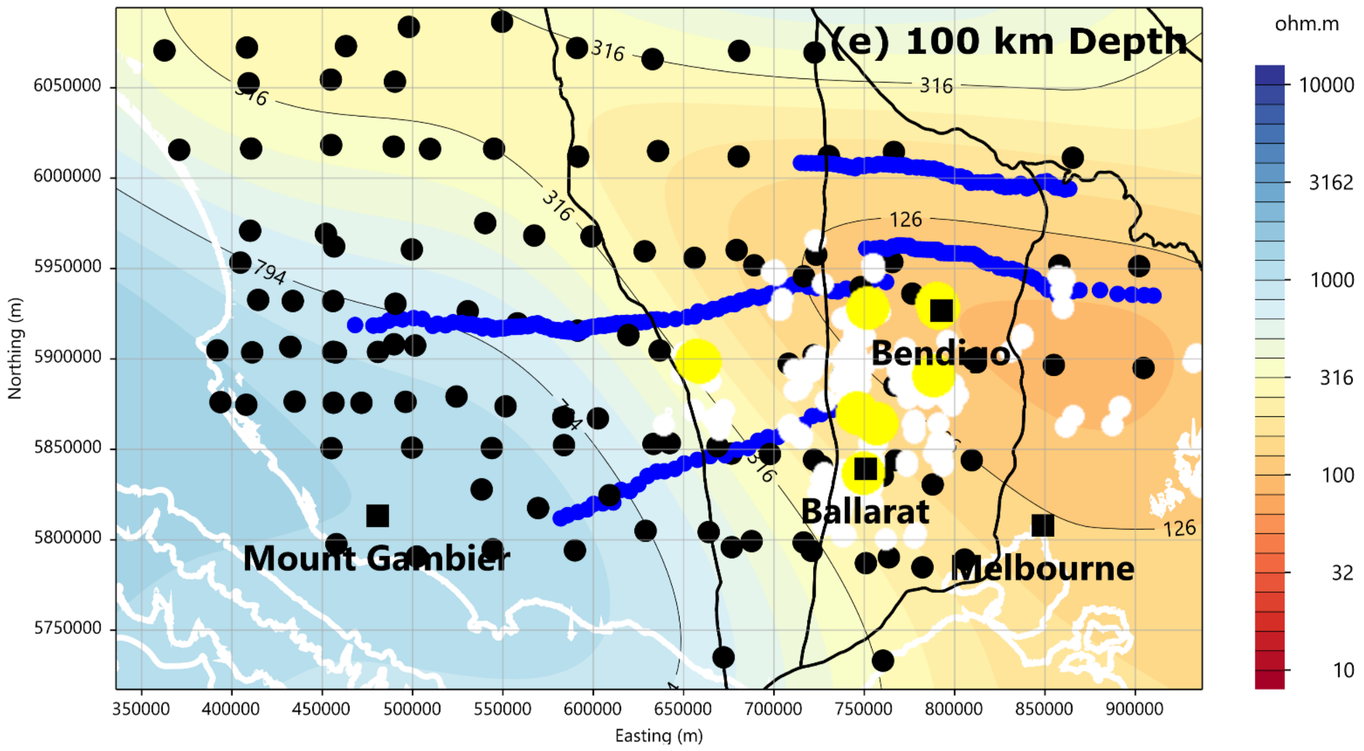

Figure S2: Depth slices of the three-dimensional model at (a) 5 km, (b) 10 km, (c) 40 km, (d) 60 km, and (e) 100 km depth. On all plots, the black dots represent long-period MT and GDS data that were used in the inversion. Blue dots are broadband MT sites that were not used in the inversion. Large red circles show significant >1 t gold deposit production, smaller red circles are < 1 t production. On the 20 km slice, real (in phase) induction arrows in the Parkinson convention are shown to clearly indicate that the three-dimensional models resistivity structures are supported by the data. Figures created using CGG Electromagnetics (Italy) Srl Geotools software (version 2.02.12400 www.cgg.com) and Inkscape (version 1.1 inkscape.org). Topography data were obtained from Geoscience Australia Geophysical Archive Data Delivery System under Creative Commons Attribution 4.0 International Licence (portal.ga.gov.au/persona/gadds).

Data fits to the preferred model are shown in Figure S3 for the phase invariant; Figure S4 for the invariant of the impedance tensor; and Figure S5 for the magnitude of the tipper. The invariants are a simple way to show quality of spatial fits at periods between 30 and 3000 s.

For phase in Figure S3, the plots shows the difference between the observed and modelled invariant with a scaling in degrees. At 30 s, there is some spatial coherence of phase misfit, but the vast majority of sites are fit to about 6° or better, which equates to an impedance error of about 10%. At longer periods of 300 and 3000, the phase misfit are generally much less than 6°.

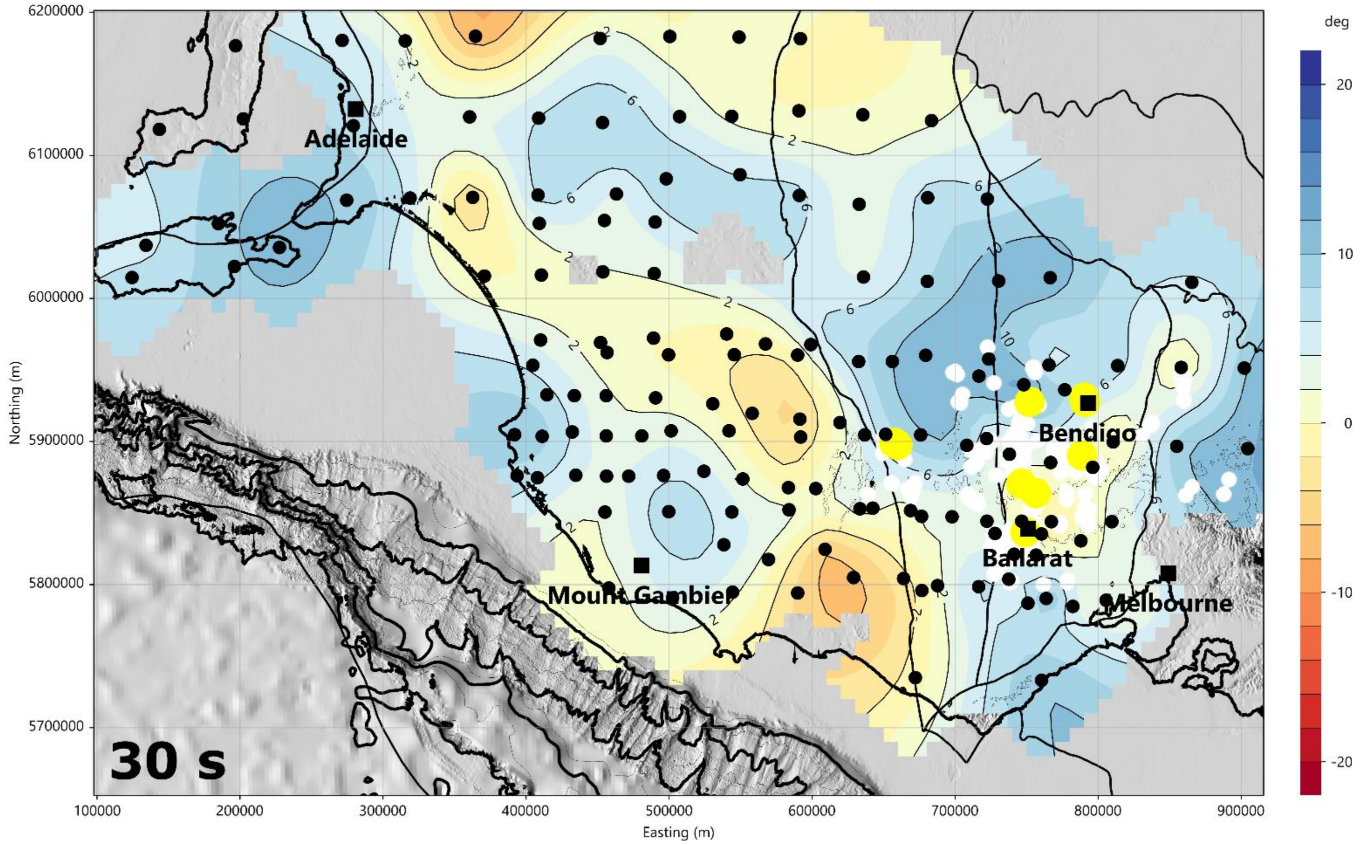

deg
Northing (m)
Easting (m)
Adelaide
Bendigo
Ballarat
Mount Gambier
Melbourne
30 s

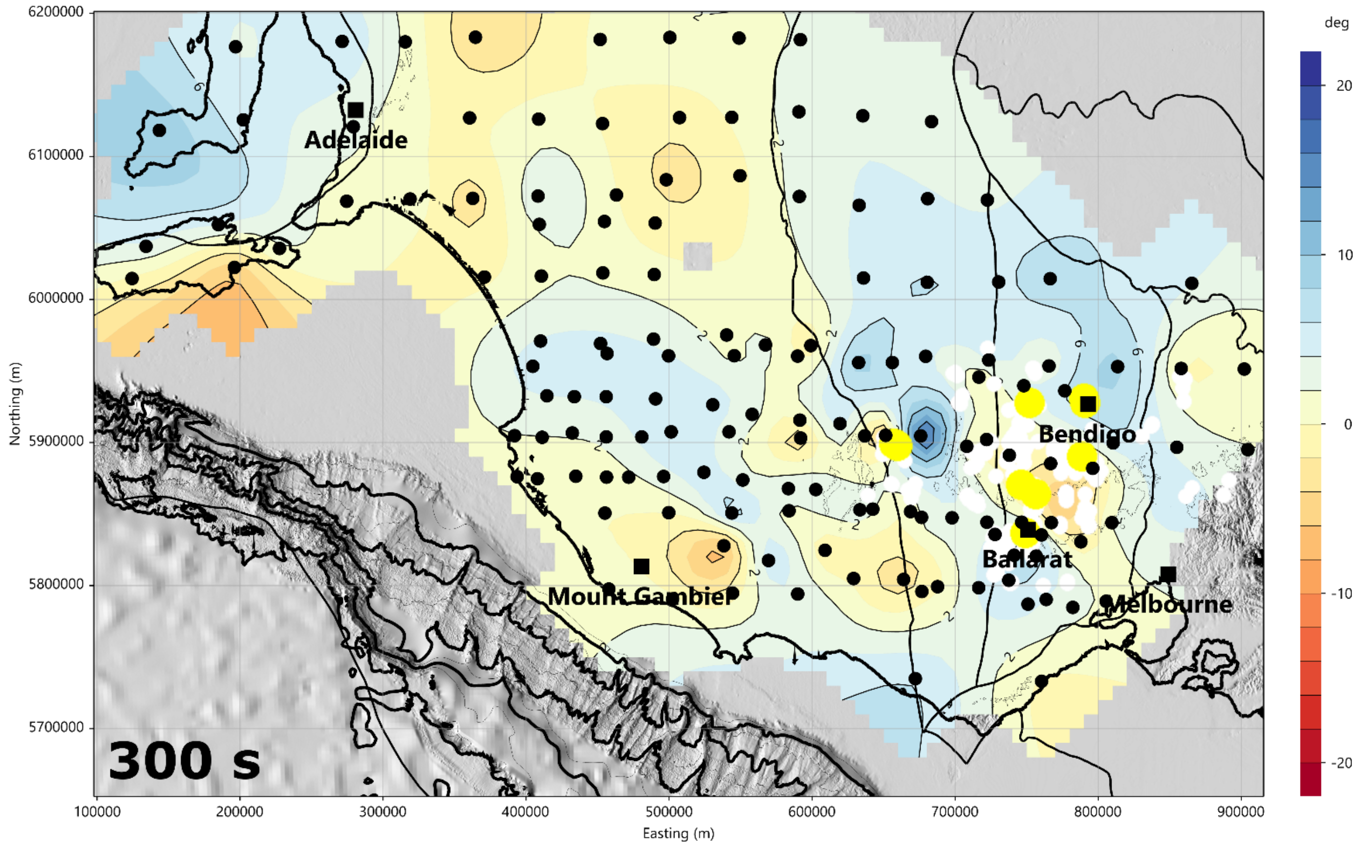

deg
Northing (m)
Easting (m)
Adelaide
Bendigo
Ballarat
Mount Gambier
Melbourne
300 s

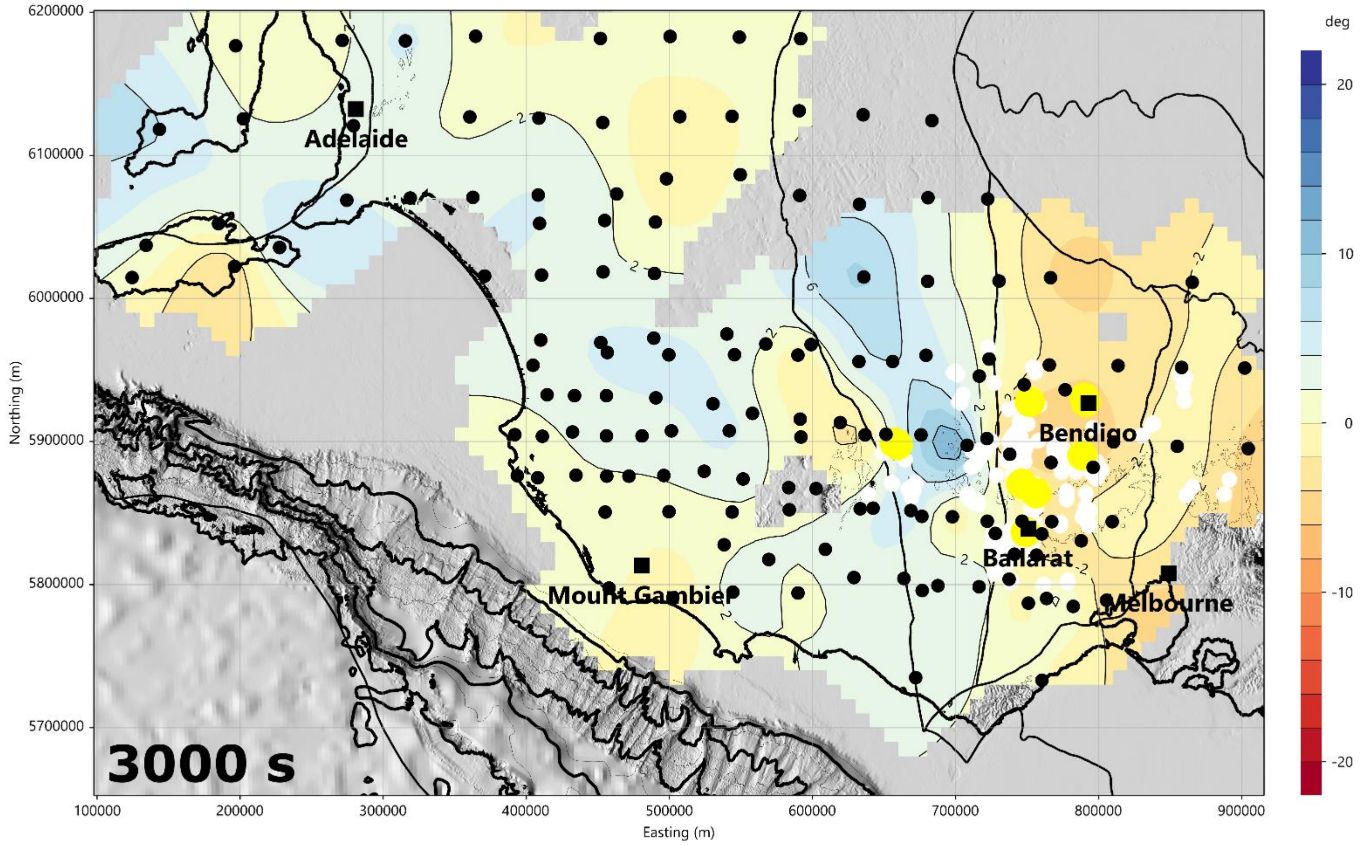

Figure S3: Normalised phase invariants (shown as the difference between the observed and modelled invariant phases), at periods of 30, 300 and 3000 s. Figures created using CGG Electromagnetics (Italy) Srl Geotools software (version 2.02.12400 www.cgg.com) and Inkscape (version 1.1 inkscape.org). Topography data were obtained from Geoscience Australia Geophysical Archive Data Delivery System under Creative Commons Attribution 4.0 International Licence (portal.ga.gov.au/persona/gadds).

Normalised invariant apparent resistivities between observed and modelled data are shown in Figure S4 on a scale of between 2 (where the observed is twice as large as the modelled value) and 0.5 (where the modelled value is twice as large as the observed); a value of 1 means the observed and modelled values are the same. There is a little more heterogeneity in the figures, with a few stations that are significantly different from neighbouring sites, producing a small bulls-eye effect. Such misfits may possible be due to small-scale distortion and static shifts that are not accommodated from the modelling. Although distortions matrices are determined in the inversions, spatial sampling of tens of kilometres and smoothing will necessarily mean site

specific distortion will still be hard to capture. Additionally, there are anthropological distortions that may affect apparent resistivity more than phase. Nevertheless, almost all sites at all periods are fit to about 20% (that is, within the range of 1.2 to 0.83) and typically less than 10%.

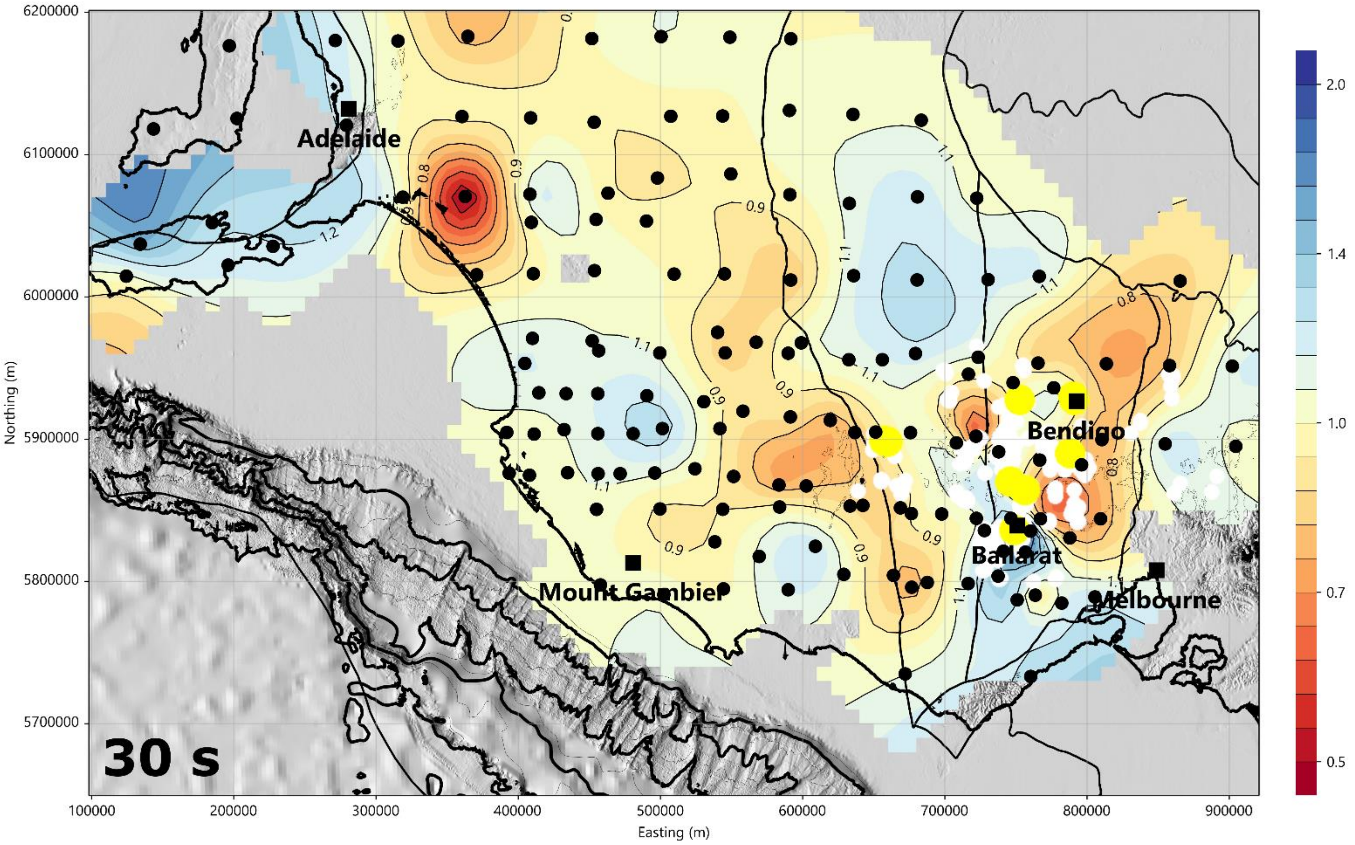
30 s
Adelaide
Bendigo
Ballarat
Mount Gambier
Melbourne
Northing (m)
Easting (m)

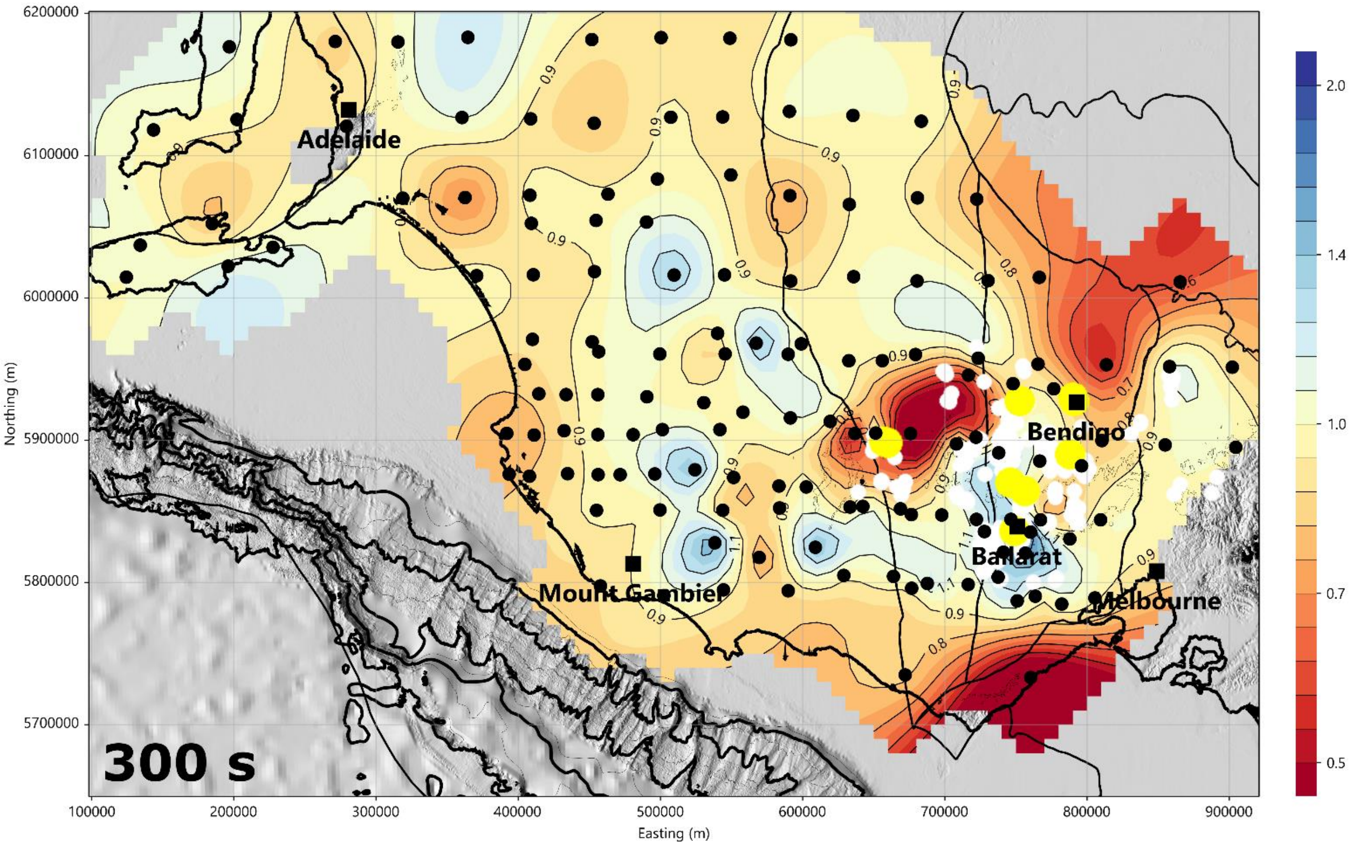
300 s
Adelaide
Bendigo
Ballarat
Mount Gambier
Melbourne
Northing (m)
Easting (m)

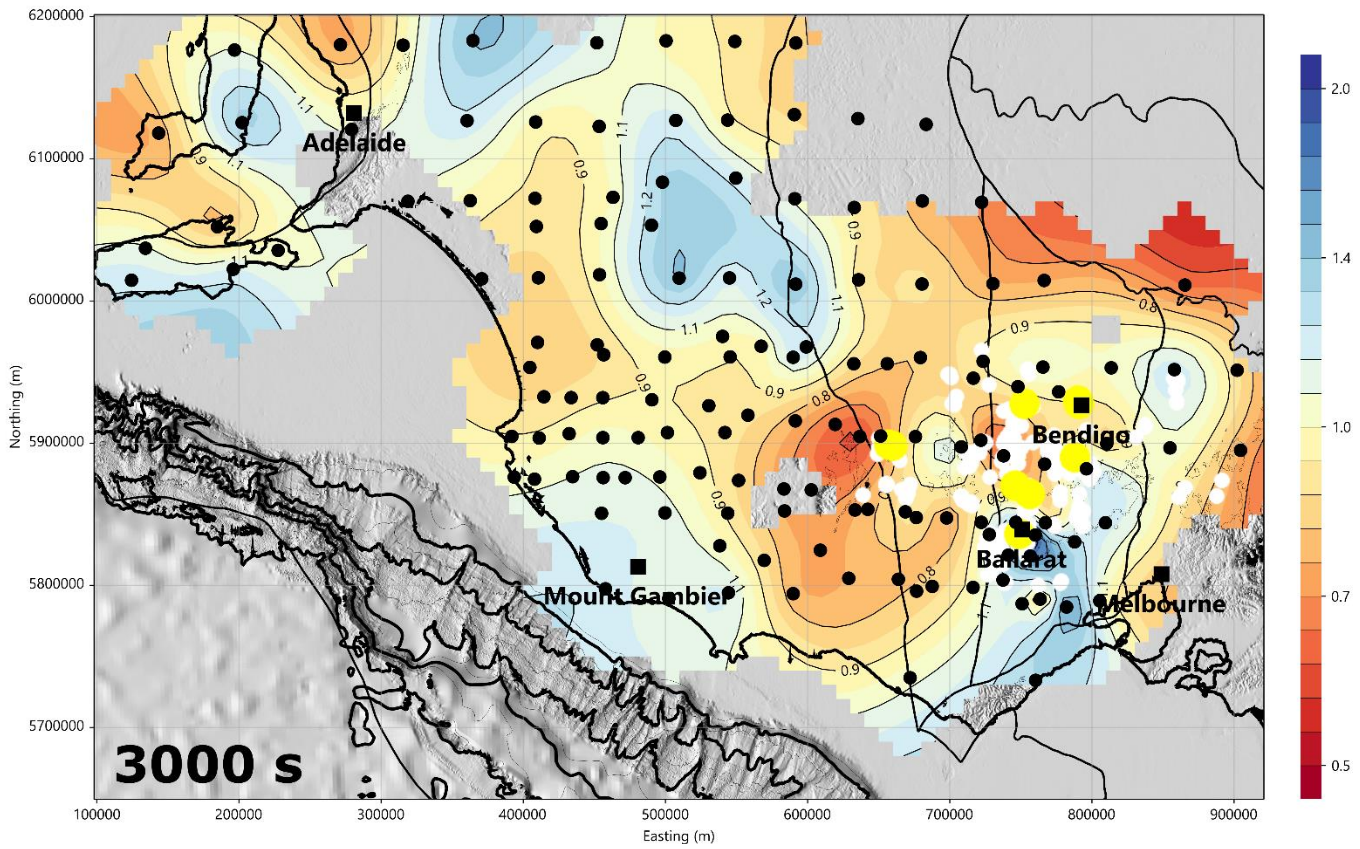

Figure S4: Normalised apparent resistivity values from the invariant of the impedance tensor (shown as the difference between the observed and modelled values, normalised by the modelled value), at periods of 30, 300 and 3000 s. A value of 2, for example, would imply the observed apparent resistivity is twice that of the modelled apparent resistivity. Figures created using CGG Electromagnetics (Italy) Srl Geotools software (version 2.02.12400 www.cgg.com) and Inkscape (version 1.1 inkscape.org). Topography data were obtained from Geoscience Australia Geophysical Archive Data Delivery System under Creative Commons Attribution 4.0 International Licence (portal.ga.gov.au/persona/gadds).

Finally, the normalised tipper magnitudes are shown in Figure S5 for periods of 100 and 1000 s. Again, there is some spatial correlation of the misfit, but generally tippers are fit to 0.05 in magnitude.

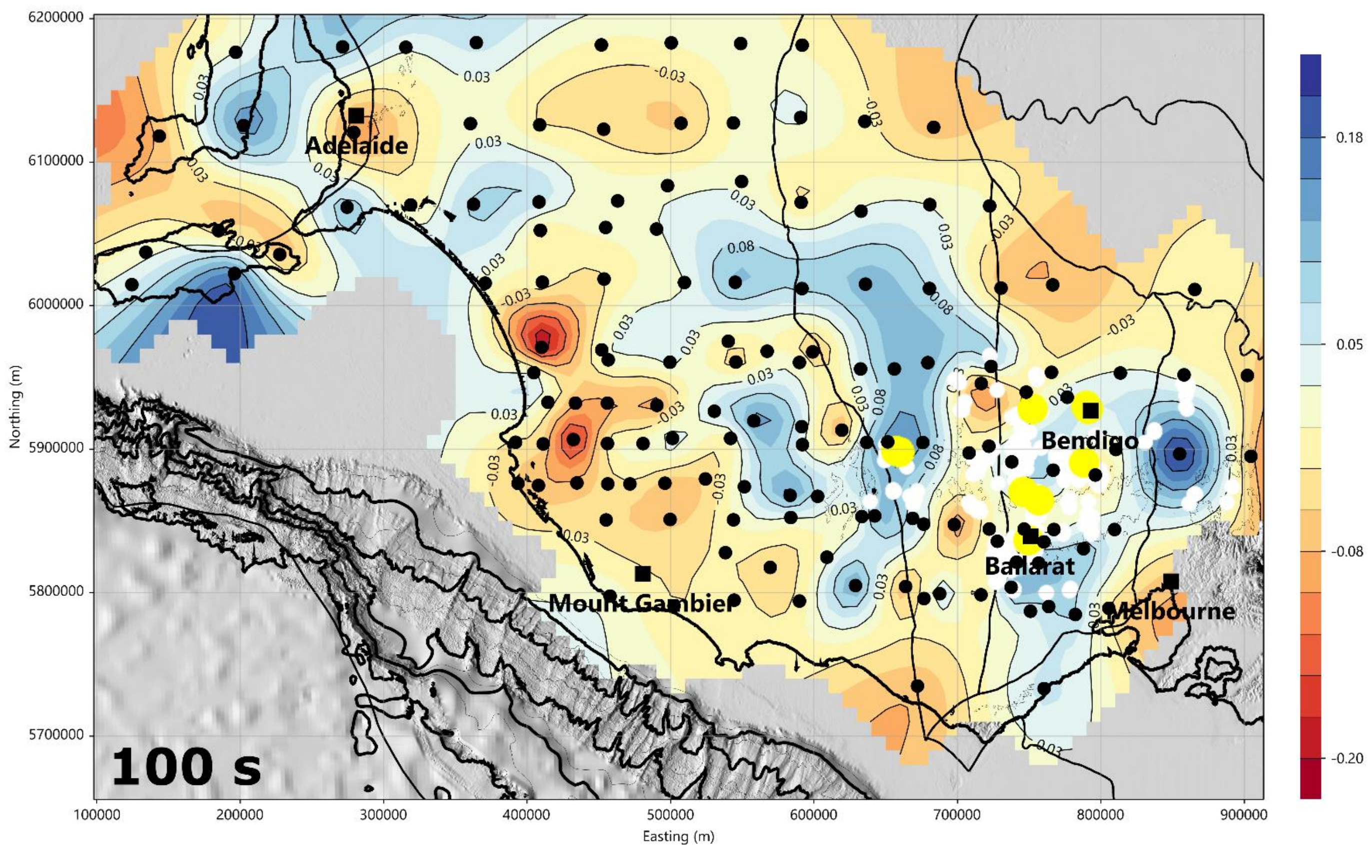

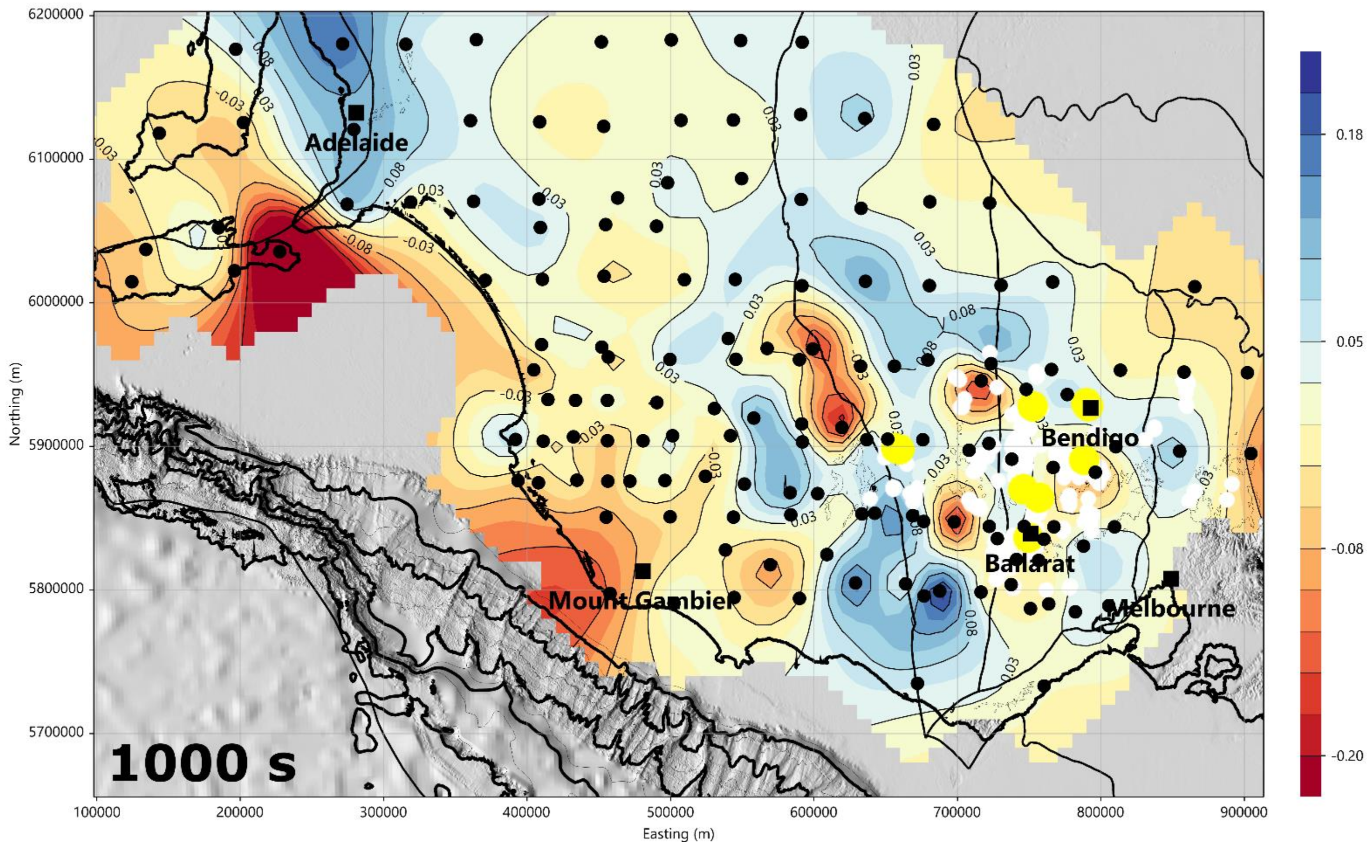

Figure S5: Normalised tipper magnitude misfits (shown as the difference between the observed and modelled tipper magnitudes), at periods of 100 and 1000 s. Figures created using CGG Electromagnetics (Italy) Srl Geotools software (version 2.02.12400 www.cgg.com) and Inkscape

(version 1.1 inkscape.org). Topography data were obtained from Geoscience Australia Geophysical Archive Data Delivery System under Creative Commons Attribution 4.0 International Licence (portal.ga.gov.au/persona/gadds).

Broadband MT Profiles

The broadband MT data have been modelled and interpreted by various authors over time, mainly in a two-dimensional context[2-6]. It was evident in the analyses that many sites exhibited three-dimensional inductive effects, but given site density along the line and bandwidth, three-dimensional inversion of profiles is complex as there are few lateral constraints away from the line of sites. We did not include broadband sites because the inversion targeted regional structures imaged by long-period data, but it is useful to demonstrate the consistency between the broadband MT sites and the three-dimensional resistivity model.

The northern MT lines[2-6] along reflection seismic profiles 06GA-V1-V3[7,8] (as shown in Figure 2) and southern Grampians lines are shown in more detail in Figure S6. Phase tensors at 30 s periods, coloured by minimum phase, and real induction arrows in the Parkinson convention at 300 s are show over the depth slice at 30 km. The northern-most line has lower minimum phase (~45-50°) indicating that the earth is not changing resistivity significantly with depth; in contrast, the middle lines have much higher minimum phase (>60°) showing that beneath these sites the resistivity is decreasing at depth. Induction arrows show as similar trend, pointing towards the most conductive region in the crust at >20 km (above 20 km the upper crust is almost uniformly resistive). Convergence of arrows along transects indicates a concentration of crustal electrical current that appears to decrease towards the north.

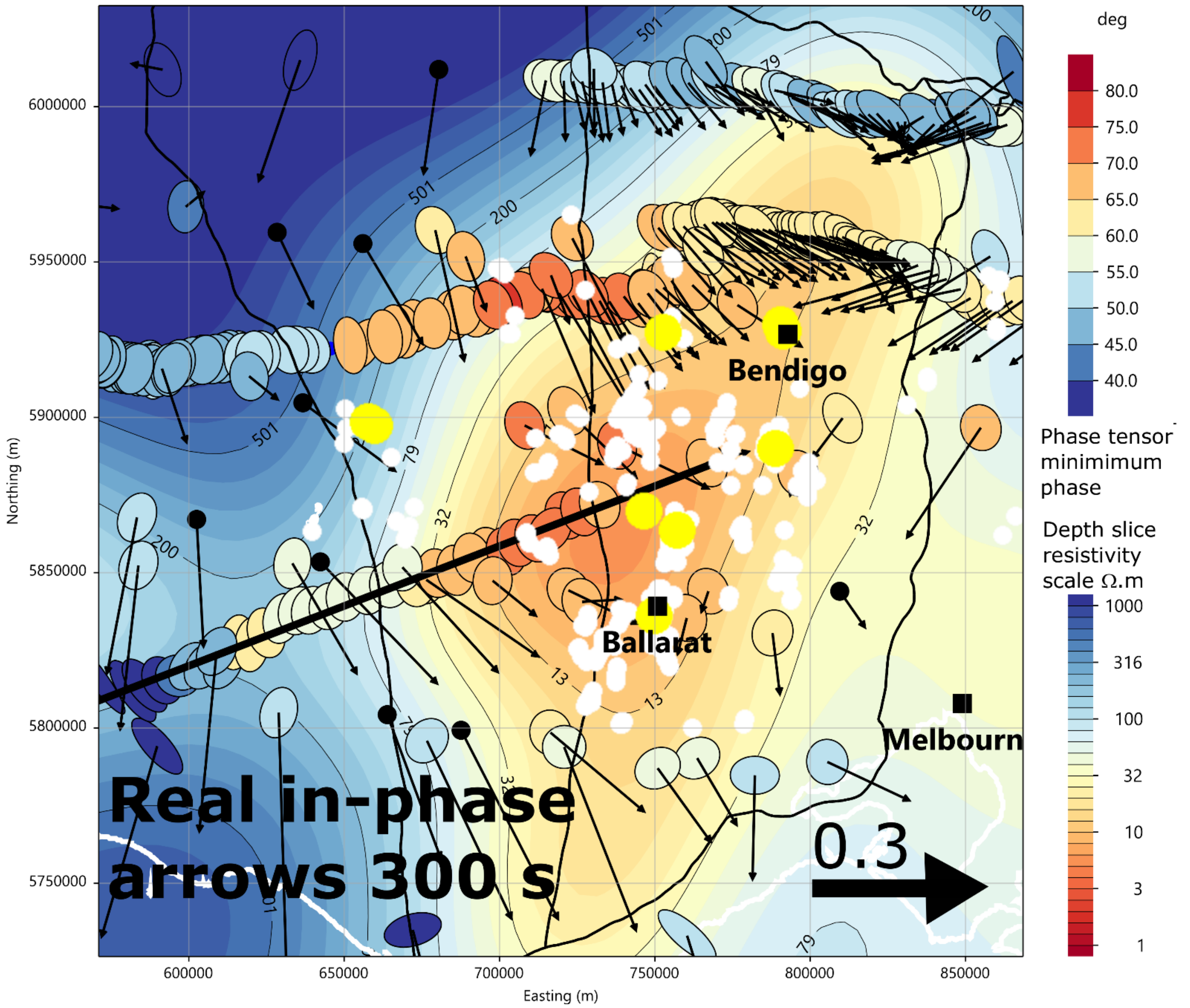

deg
80.0
75.0
70.0
65.0
60.0
55.0
50.0
45.0
40.0
Phase tensor minimimum phase
Depth slice resistivity scale Ω.m
1000
316
100
32
10
3
1
Northing (m)
6000000
5950000
5900000
5850000
5800000
5750000
Easting (m)
600000
650000
700000
750000
800000
850000
Bendigo
Ballarat
Melbourn
Real in-phase arrows 300 s
0.3

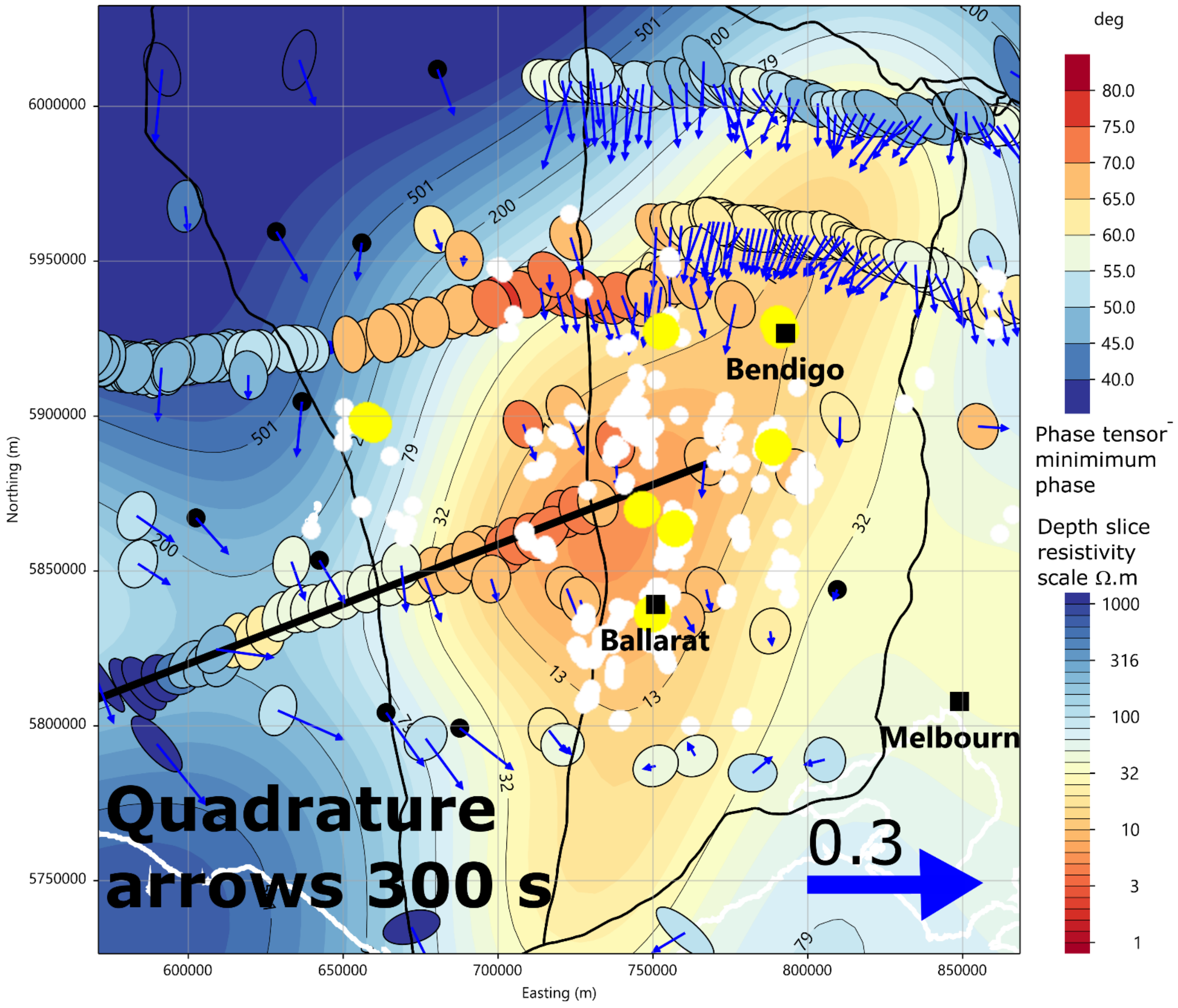

Figure S6: Broadband MT lines (densely located sites) and regional long-period MT sites plotted on the resistivity model at 30 km depth. The Grampians MT line is shown by the thick black line. Phase tensors at a period of 30 s are coloured with minimum phase, with red colours indicating decreasing resistivity with depth, and blue colours showing less change. Real (in-phase) induction arrows at a period of 300 s are shown the Parkinson convention in top figure; quadrature arrows are shown in the lower figure. Gold deposits (large yellow circles >1 t production, and small white circles <1 t), mostly are within the 20 Ω.m contour of resistivity at 30 km. Figure created using CGG Electromagnetics (Italy) Srl Geotools software (version 2.02.12400 www.cgg.com) and Inkscape (version 1.1 inkscape.org).

Modelling and interpretation of the broadband MT along seismic lines 06GA-V1-V3 have been published. The only unpublished broadband MT survey was collected along the Grampians line in 2015 by the University of Adelaide and the Geological Survey of Victoria. Thirty-three sites of data were collected along a profile, as shown in Figure S7. The overall profile length was ~160 km long with site spacing of about 5 km. Data were collected with AuScope broadband MT instruments, and produced estimates in the bandwidth of 100 Hz to 1000 s. Vertical magnetic induction B_z component was not recorded due to the time-logistics of deployment. Figure S3 shows a strong correlation between the Grampians line MT data and the three-dimensional model at a depth of 30 km. Sites at the western end, with low minimum phase (blue colours) are due to their location at the margin of the Otway Basin, and thus reflect the increasing resistivity from porous sediments to upper crust.

Dimensionality tests of skew <5 ^{o9} suggests that most sites are compatible with a two-dimensional earth to a period of about 30 s. We therefore undertook a 2D inversion of the broadband MT data in the bandwidth 100 Hz to 10 s for smooth resistivity structure. The inversion used only the new broadband MT responses and thus represents an independent image. The inversion comprised 57785 cells to a depth of 200 km. After a number of trials, smoothing weight was set at 0.1 and the horizontal and vertical gradients were given the same weighting. After rotating to an angle of 335°, both TE and TM responses were inverted with a 5% error floor on the impedance (10% on apparent resistivity, 5% on phase), and the fit has an overall RMS of 2.0. Static shifts were included in the inversion. Figure S7a shows the smooth two-dimensional model from the Grampians line, and Figure S7b shows the corresponding profile extracted from the three dimensional inversion. Model fits for the two-dimensional inversion are shown in Figure S8.

The two profiles are remarkably similar given that they were generated from two entirely different data sets, with different spatial site intervals, and bandwidth. The key feature on each is the low-resistivity (~20 Ω.m) region at 20-40 km depth on the eastern side of the model, and the overlying resistive (>1000 Ω.m) in the upper crust, which has been identified as the orogenic source of the gold deposits. Also clearly defined are the conductive sediments in the top 5 km at the far western end of the profile. Below the Moho, both

models indicate that there is a gradient in resistivity in the mantle towards the east, but the two-dimensional model lacks resolution as the longest period of 100 s is significantly less than the 10000 s in the three dimensional model.

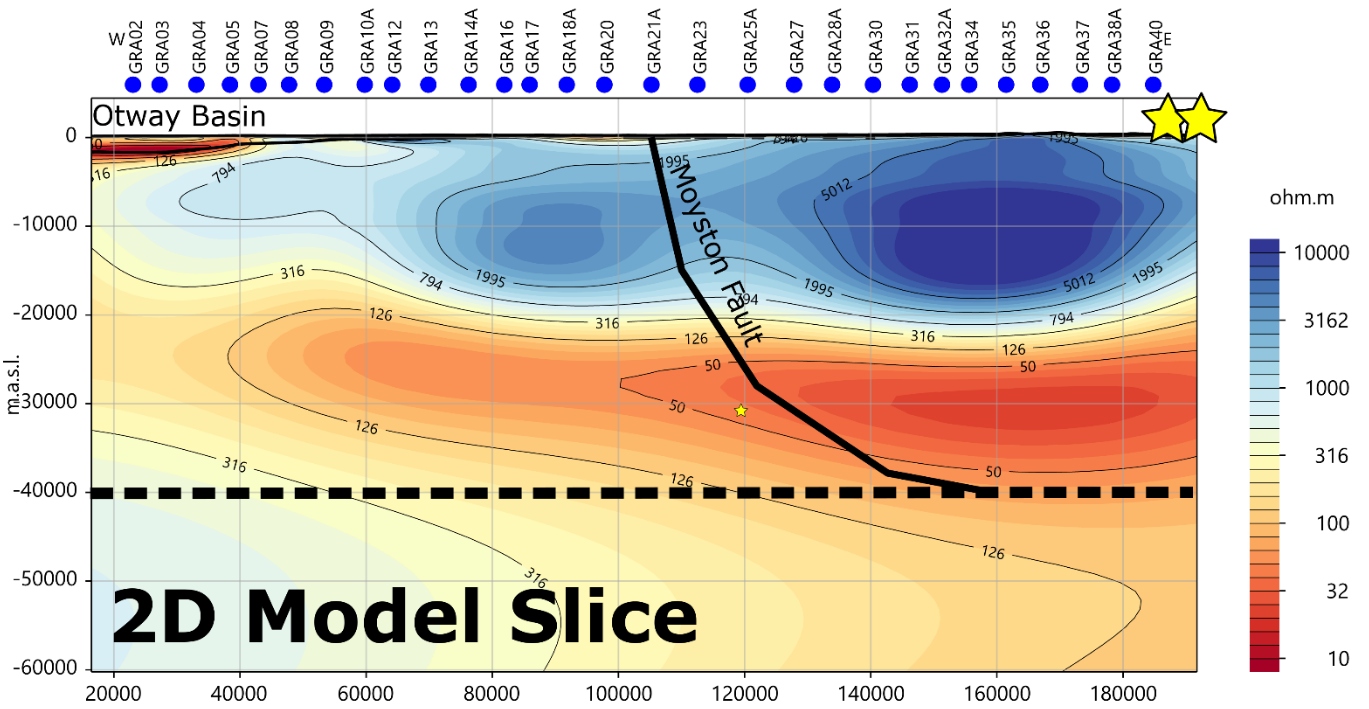

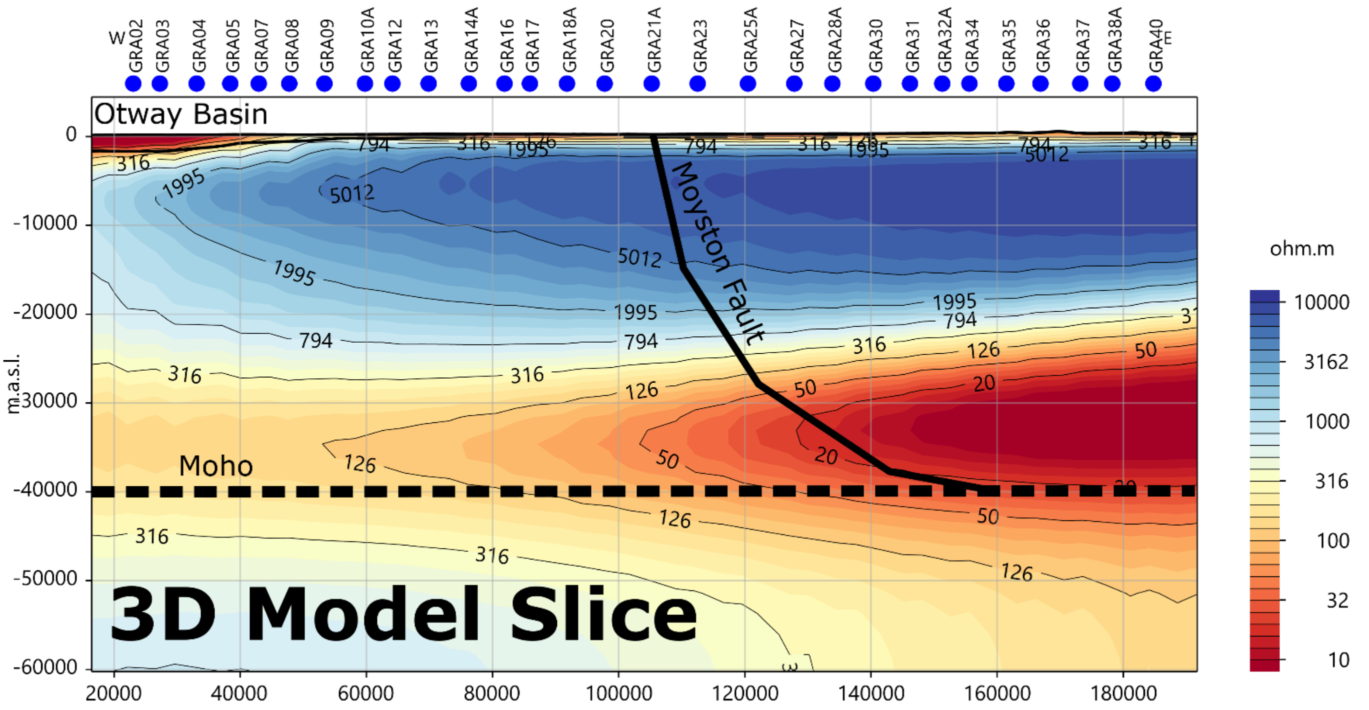

Figure S7: The upper figure shows a two-dimensional inversion of thirty three broadband MT sites, spaced ~5 km apart, in the bandwidth of 100 Hz to 10 s. The approximate depth of the Moho, and location of the Moyston Fault are shown; the dip on the Moyston Fault is inferred from the dip in seismic section 06GA-V1 but is not known specifically for this location. Gold stars show location of > 1T gold deposits adjacent the profile line. The lower figure shows the same profile, but extracted from the three-dimensional model. In this case, the model is mostly defined by AusLAMP long-period MT sites spaced 50 km apart, with a few additional legacy long-period MT and GDS site, in the bandwidth of 10 to 10000 s. Despite the differences in data provenance and non-overlapping bandwidth of frequencies, the models are remarkably similar in terms of depths, resistivities and broad-scale structure. Figure created using CGG Electromagnetics (Italy) Srl Geotools software (version 2.02.12400 www.cgg.com) and Inkscape (version 1.1 inkscape.org).

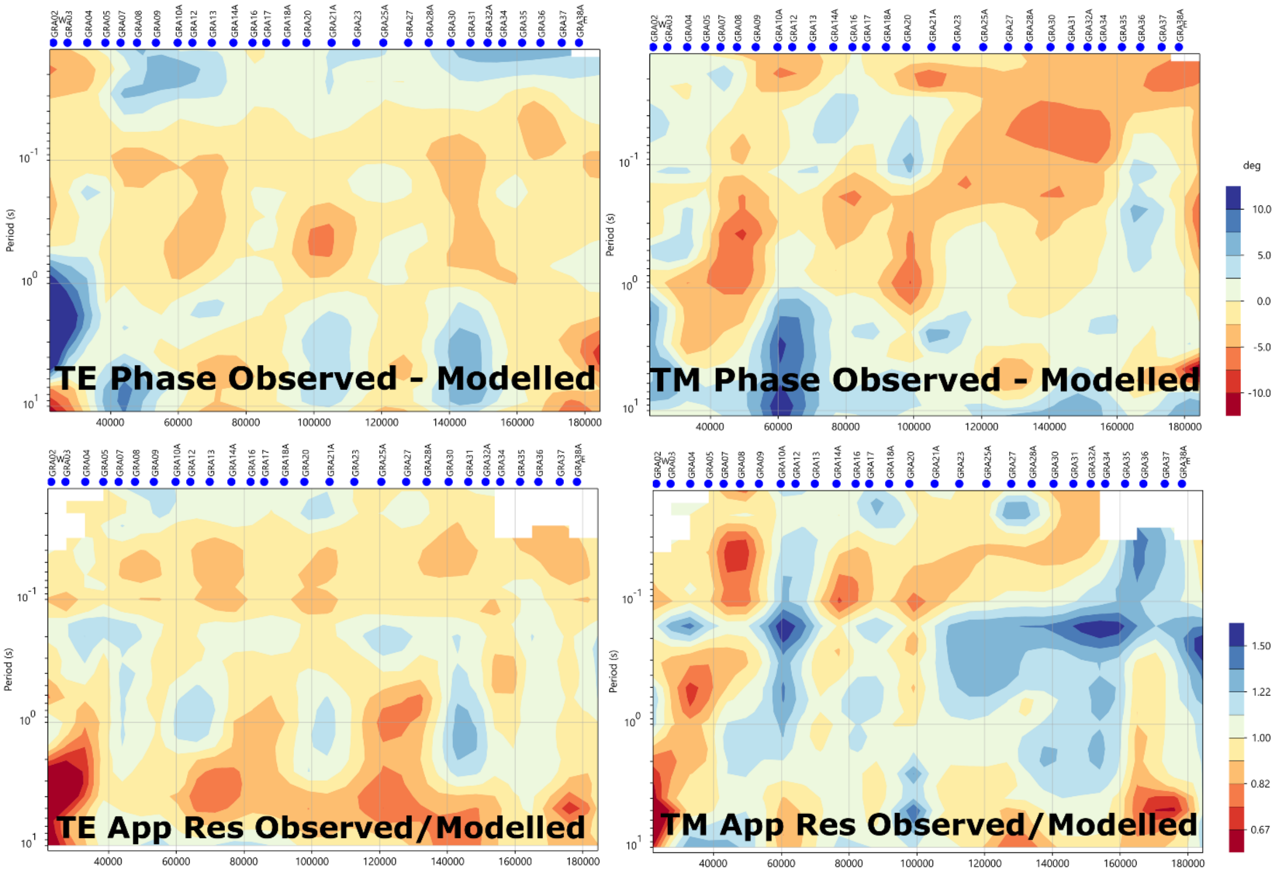

Figure S8: Normalised pseudo-sections of the apparent resistivity and phase in TE and TM modes. Figure created using CGG Electromagnetics (Italy) Srl Geotools software (version 2.02.12400 www.cgg.com) and Inkscape (version 1.1 inkscape.org).

References

1 Cas, R. A. F., van Otterloo, J., Blaikie, T. N. & van den Hove, J. in *Geological Society Special Publication* Vol. 446 123-172 (2017).

2 Dennis, Z. R., Moore, D. H. & Cull, J. P. A geological interpretation of the Echuca magnetotelluric survey, Victoria. *Australian Journal of Earth Sciences* **58**, 587-597, doi:10.1080/08120099.2011.577808 (2011).

3 Dennis, Z. R., Moore, D. H. & Cull, J. P. Magnetotelluric survey for undercover structural mapping, Central Victoria. *Australian Journal of Earth Sciences* **58**, 33-47, doi:10.1080/08120099.2011.534817 (2011).

4 Dennis, Z. R., Thiel, S. & Cull, J. P. Lower crust and upper mantle electrical anisotropy in southeastern Australia. *Exploration Geophysics* **43**, 228-241, doi:10.1071/EG11022 (2012).

5 Robertson, K., Taylor, D., Thiel, S. & Heinson, G. Magnetotelluric evidence for serpentinisation in a Cambrian subduction zone beneath the Delamerian Orogen, southeast Australia. *Gondwana Research* **28**, 601-611, doi:10.1016/j.gr.2014.07.013 (2015).

6 Robertson, K. E., Heinson, G. S., Taylor, D. H. & Thiel, S. The lithospheric transition between the Delamerian and Lachlan orogens in western Victoria: new insights from 3D magnetotelluric imaging. *Australian Journal of Earth Sciences* **64**, 385-399, doi:10.1080/08120099.2017.1292953 (2017).

7 Cayley, R. A. *et al.* Crustal architecture of central Victoria: Results from the 2006 deep crustal reflection seismic survey. *Australian Journal of Earth Sciences* **58**, 113-156, doi:10.1080/08120099.2011.543151 (2011).

8 Willman, C. E. *et al.* Crustal-scale fluid pathways and source rocks in the victorian gold province, Australia: Insights from deep seismic reflection profiles. *Economic Geology* **105**, 895-915, doi:10.2113/gsecongeo.105.5.895 (2010).

9 Caldwell, T. G., Bibby, H. M. & Brown, C. The magnetotelluric phase tensor. *Geophysical Journal International* **158**, 457-469, doi:10.1111/j.1365-246X.2004.02281.x (2004).
